# Key features and homing properties of NK cells in the liver are shaped by activated iNKT cells

**DOI:** 10.1038/s41598-019-52666-9

**Published:** 2019-11-08

**Authors:** Stephanie Trittel, Benedict J. Chambers, Ulrike Heise, Carlos A. Guzmán, Peggy Riese

**Affiliations:** 1 Department of Vaccinology and Applied Microbiology, Helmholtz Centre for Infection Research, D-38124 Braunschweig, Germany; 2 Center for Infectious Medicine, Department of Medicine, Karolinska Institute, Karolinska University Hospital Huddinge, Stockholm, S-14186 Sweden; 3 Department of Mouse Pathology, Helmholtz Centre for Infection Research, D-38124 Braunschweig, Germany

**Keywords:** Viral infection, Innate immune cells

## Abstract

The contribution of natural killer (NK) cells to the clearance of hepatic viral infections is well recognized. The recently discovered heterogeneity of NK cell populations renders them interesting targets for immune interventions. Invariant natural killer T (iNKT) cells represent a key interaction partner for hepatic NK cells. The present study addressed whether characteristics of NK cells in the liver can be shaped by targeting iNKT cells. For this, the CD1d-binding pegylated glycolipid αGalCerMPEG was assessed for its ability to modulate the features of NK cells permanently or transiently residing in the liver. *In vivo* administration resulted in enhanced functionality of educated and highly differentiated CD27^+^ Mac-1^+^ NK cells accompanied by an increased proliferation. Improved liver homing was supported by serum-derived and cellular factors. Reduced viral loads in a mCMV infection model confirmed the beneficial effect of NK cells located in the liver upon stimulation with αGalCerMPEG. Thus, targeting iNKT cell-mediated NK cell activation in the liver represents a promising approach for the establishment of liver-directed immune interventions.

## Introduction

Natural killer (NK) cells belong to the innate lymphocyte population and represent an essential component of the first line of defense against virally infected and transformed cells^1^. Additionally, they exert regulatory functions by secreting a variety of cytokines critical for innate and adaptive immune responses^2–4^. New insights into the mechanisms controlling NK cell functionality emphasize their heterogeneity and render them potential targets for immune interventions^5–7^. However, it is critical to expand our knowledge on organ-specific features using appropriate preclinical models in order to evolve therapeutic strategies targeting NK cells. Mouse and human NK cell subsets display a number of similarities rendering the murine model valid for studying human NK cell biology^8,9^.

Murine NK cell differentiation is described as a sequential process determined by the expression of CD27 and Mac-1^10–12^. CD27^high^ Mac-1^low^ NK cells represent an immature phenotype with a large proliferative capacity. NK cells expressing both CD27 and Mac-1 display a low activation threshold and account for the most functional subset holding the highest cytotoxic and cytokine secreting potential. Terminally differentiated CD27^low^ Mac-1^high^ NK cells have a considerably higher activation threshold rendering them less responsive as compared to the CD27^high^ Mac-1^high^ cell subset. Human blood-derived NK cells can also be classified by CD27 and Mac-1 expression, although here the classification into CD56^bright^ and CD56^dim^ is more explicit^13,14^. The most functional CD27^high^ Mac-1^high^ NK cell subset possesses an increased expression of the spleen exit marker CXCR3, thereby showing an enhanced migration potential as compared to CD27^low^ Mac-1^high^-expressing NK cells^10^. Thus, NK cells harbor a differential migration capacity according to their differentiation status.

The ability of NK cells to become functionally active is also controlled by a process termed “education” that enables selective distinguishing between self and non-self cells to ensure self-tolerance^15–17^. Educated NK cells expressing inhibitory receptors of the Ly49C, Ly49I family and CD94/NKG2A, bind to self-MHC class I molecules and thereby become functional when activating signals override inhibitory signals. Uneducated NK cells do not express the cognate ligand for self-MHC class I molecules and are thus considered as hypo-responsive^5,18^.

Most of the studies, which investigated NK cells under healthy and diseased conditions, were focused on blood- or lymphoid tissue-derived NK cells. However, NK cells depict organ specific characteristics with quite distinct phenotypes and functional features^19^. Here, the liver contains the highest percentage of NK cells (10–20% in mice/25–40% in humans) under steady state amongst all organs^20^. Recent reports state a developmental process of hepatic NK cells different from splenic NK cells^21–23^. Under steady state conditions, splenic NK cells display a more mature phenotype, whereas hepatic NK cells are rather immature. Various liver-specific cell types, such as Kupffer cells and liver sinusoidal endothelial cells, shape a unique microenvironment in close connection with liver-resident and circulating immune cell populations. The continuous exposure to antigens and microbial products leads to an altered responsiveness of liver-resident cells keeping them in a state termed “active tolerance”^24^. Hence, hepatic NK cells contribute to immune tolerance while at the same time inducing immune responses against invading pathogens^25^. However, under inflammatory or infectious conditions, not only liver-resident but also conventional, circulating NK cells transiently residing in the liver contribute crucially to local immune responses^26–28^. Thus, both populations are essential for optimal hepatic immunity.

NK cell functionality is known to correlate with the resolution of hepatic infections and malignancies in a variety of clinical settings. The specific modulation of NK cell features might represent a powerful tool for the initiation of protective intrahepatic immune responses. Besides NK cells, the liver harbors the largest percentage of invariant natural killer T (iNKT) cells as compared to any other organ^29^. Invariant NKT cells can become activated by the glycolipid α-galactosylceramide (αGalCer), presented by CD1d which is expressed by various lymphoid and non-lymphoid cell types^30–32^. Several clinical trials investigating the therapeutic potential of αGalCer-pulsed dendritic cells against cancer suggest a beneficial effect, mainly caused by IFNγ induction^33–37^. However, clinical studies addressing the antiviral activity of αGalCer against hepatitis were less successful^38,39^.

A pegylated derivative of αGalCer (αGalCerMPEG) was demonstrated to have improved biological and functional properties including a higher potency and improved solubility in water as compared to the parental compound^40^. Recently, we have shown that αGalCerMPEG-activated iNKT cells have a beneficial impact on the differentiation and education status of splenic NK cells resulting in the generation of highly functional NK cells with improved antiviral activity^41^.

The potential to fine-tune NK cell functionality in a desired direction at the unique site of the liver might represent a big step forward in the establishment of immune interventions against infectious liver diseases. We therefore addressed whether permanently and transiently liver-resident NK cells can be modulated by the iNKT cell agonist αGalCerMPEG.

## Results

### αGalCerMPEG-mediated activation of NK cells in the liver

**Figure 1:**
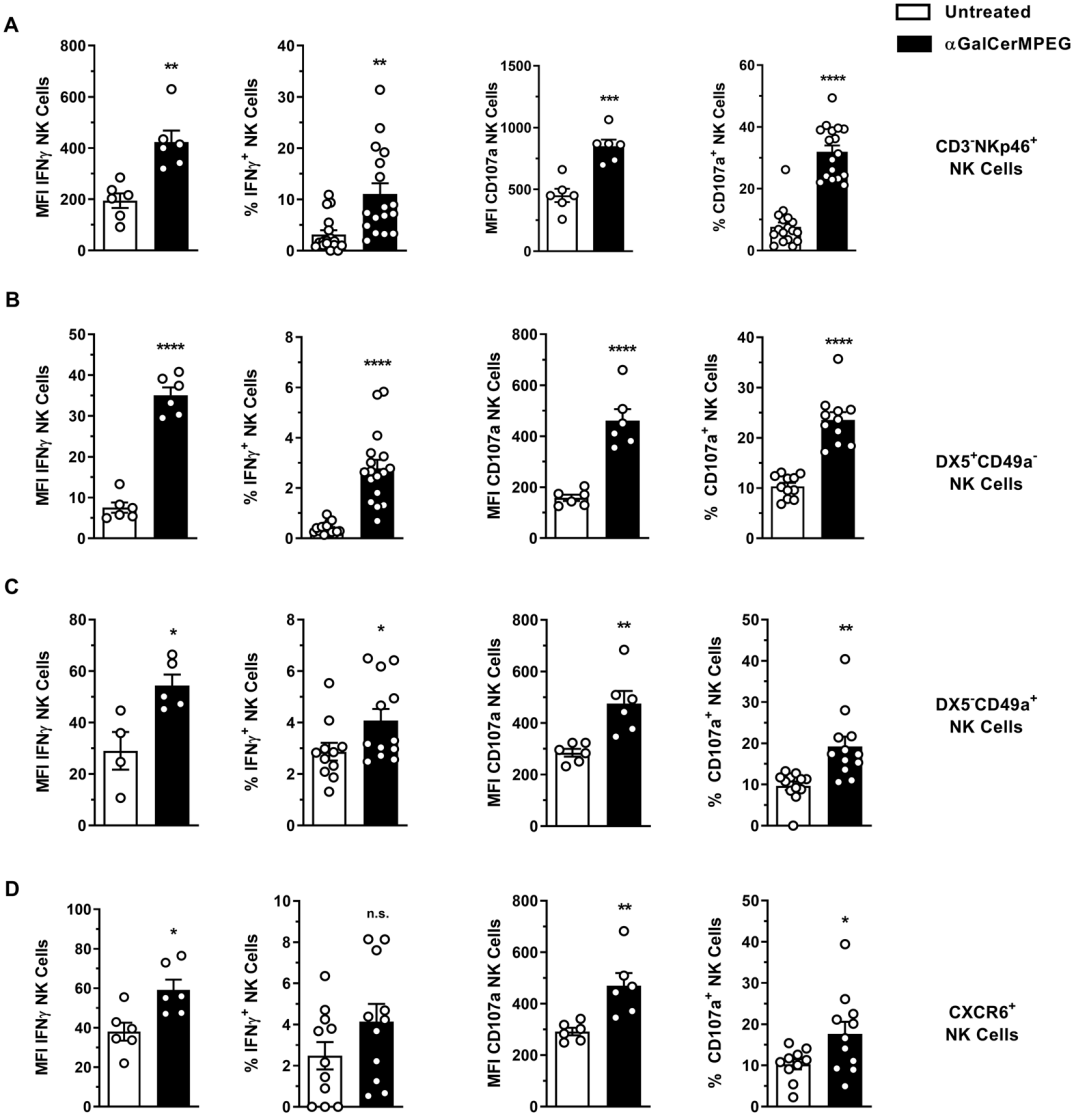
Improved hepatic NK cell activation, cytokine secretion and cytotoxicity following iNKT cell stimulation. Wild type (wt) mice were injected by s.c. route with a single dose of αGalCerMPEG (10 µg). Liver-derived lymphocytes were isolated 72 h after administration. NK cell populations (NKp46^+^CD3^−^, DX5^+^CD49a^−^, DX5^−^CD49a^+^ and CXCR6^+^) were stained for the expression of IFNγ and CD107a following 6 h co-culture with YAC-1 target cells. MFI and frequencies of (**A**) CD3-NKp46^+^, (**B**) DX5^+^CD49a^−^, (**C**) DX5^−^CD49a^+^ and (**D**) CXCR6^+^ NK cells isolated from wt mice expressing IFNγ and CD107a (MFI: n = 3–6 mice, one out of two or more independent representative experiments, Frequencies: n = 6–17 mice). Columns represent the mean ± SEM and circles indicate single values. Asterisks denote significant values as calculated by unpaired, two-tailed Student’s t-test. ****p ≤ 0.0001; ***p ≤ 0.001; **p ≤ 0. 01; *p ≤ 0; 05; n.s. = not significant.

A beneficial effect of αGalCerMPEG on splenic NK cell functionality, *such as* enhanced CD69 expression, IFNγ secretion and degranulation capacity was recently demonstrated by our group^41^. Thus, the potential of subcutaneously (s.c.)-administered αGalCerMPEG to induce activation of NK cells located in the liver at the time of analysis (including tissue-resident as well as circulating/recruited NK cells) was assessed. To this end, NK cells isolated from the liver 72 h after stimulation were co-incubated with YAC-1 target cells and analyzed for NK cell activation and functionality. Like splenic NK cells, CD3^−^NKp46^+^ NK cells displayed a significantly enhanced activation status and an improved responsiveness as indicated by an elevated secretion of IFNγ and enhanced up-regulation of CD107a and CD69 as compared to untreated controls. Additionally, increased frequencies of IFNγ-secreting and degranulating NK cells were detected (Figs 1A, S1 and S3C). Next to the spleen and liver, a αGalCerMPEG-mediated NK cell activation was also detected in the blood, lymph nodes (LN), lung and intraperitoneal adipose tissue (AT) (Fig. S2). Trafficking conventional NK cells (NKp46^+^CD3^−^) were shown to express DX5 and lack the expression of CD49a, permanently liver-resident NK cells on the other hand were recently described as DX5^−^CD49a^+^ or CXCR6-expressing NK cells^28,42^. Here, enhanced αGalCerMPEG-mediated activation and functionality of DX5^+^CD49a^−^ as well as DX5^−^CD49a^+^ and CXCR6^+^ NK cells isolated from the liver were detected with regard to the expression density and frequencies of IFNγ and CD107a (Fig. 1B–D).

The treatment of NKT cell-deficient Jα281^−/−^ mice with αGalCerMPEG did not result in any alteration with respect to NK cell activation and functionality as compared to untreated controls, although wt and Jα281^−/−^ mice harbor similar NK cell frequencies under steady state (Fig. S3). These findings confirm the necessity of iNKT cells for αGalCerMPEG-mediated NK cell stimulation in the liver.

The analysis of absolute hepatic iNKT cell numbers revealed an increase upon αGalCerMPEG administration, especially of those ascribed to the NKT1 cell population (Fig. S4)^43,44^. Here, especially those NKT cells characterized by CD4^+^T-bet^+^ or IL-17RB^−^ were significantly activated to produce IFNγ, IL-4 and IL-17.

The comparison of αGalCerMPEG with the parental compound αGalCer revealed a superiority of the pegylated derivative concerning the activation of NK cells, despite a 33-fold lower amount of the biological active substance αGalCer^40^. In accordance with these observations, administration of αGalCerMPEG induced significantly increased frequencies of IFNγ-secreting and CD107a-expressing NK cells in the liver as compared to the parental compound αGalCer (Fig. S5).

The assessment of the education status revealed that iNKT cell stimulation by αGalCerMPEG led to the activation of educated rather than uneducated NK cells in the liver. The administration of αGalCerMPEG resulted in elevated frequencies of IFNγ-secreting and CD107a-expressing educated NK cells as compared to uneducated NK cells and untreated controls. Educated NK cells further showed an increased expression density of CD107a (Fig. S6A). These findings indicate that iNKT cell activation by αGalCerMPEG leads to the generation of highly active educated NK cells in the liver.

### Invariant NKT cell activation induces the accumulation of functional mature NK cells in the liver

**Figure 2:**
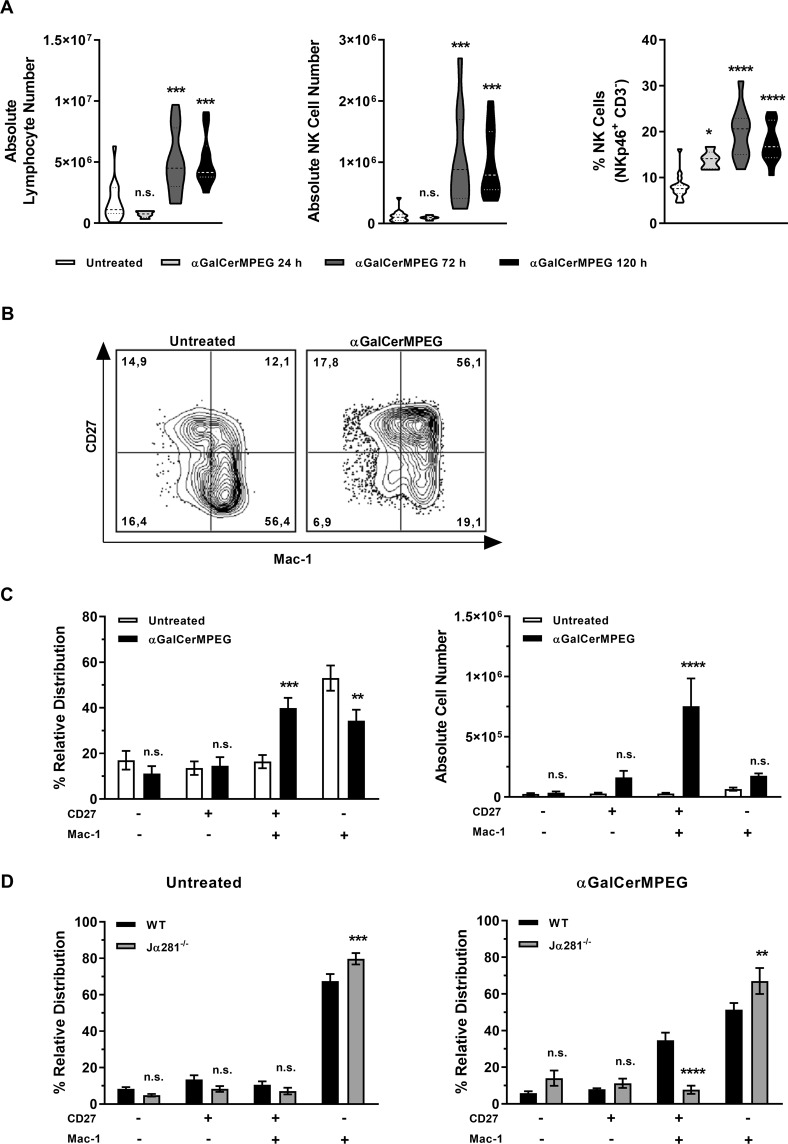
iNKT cell-mediated generation of highly mature NK cell subsets in the liver. Hepatic lymphocytes derived from wt and iNKT cell-deficient Jα281^−/−^ mice were isolated at the indicated time points after s.c. administration of a single dose of αGalCerMPEG (10 µg). (**A**) Absolute numbers of hepatic lymphocytes (out of 1 × 10^6^ total cells), NK cells (NKp46^+^CD3^−^) and frequencies of NK cells (n = 5–11 mice). Boxes represent the interquartile range, horizontal lines show the mean value and whiskers show the overall range of the data. (**B**) Representative FACS staining for CD27- and Mac-1-expressing hepatic NK cells in untreated and treated animals. (**C**) Relative distribution (n = 14–16 mice) and absolute cell numbers (out of 1 × 10^6^ total cells, n = 9–11 mice) of CD27- and Mac-1-expressing NK cells isolated from wt mice. (**D**) Relative distribution of NK cells isolated from untreated and treated wt and NKT cell-deficient Jα281^−/−^ mice (n = 7 mice). Violin plots represent the interquartile range, horizontal lines show the mean value and the width displays the distribution of data points. Columns represent the mean ± SEM of data pooled from two or more independent experiments. Asterisks denote significant values as calculated by One-way (**A**) or Two-way ANOVA (**C**,**D**) as compared to untreated controls. ****p ≤ 0.0001; ***p ≤ 0.001; **p ≤ 0.01; *p ≤ 0.05; n.s. = not significant.

To investigate whether the increased functionality of liver NK cells is associated with changes in absolute hepatic cell numbers, the absolute lymphocyte and NK cell numbers were assessed following s.c. administration of αGalCerMPEG. Significantly elevated numbers of both lymphocytes and NK cells were observed 72 h after iNKT cell stimulation, whereas NK cell frequencies were already significantly increased after 24 h (Fig. 2A). The analysis of NK cell populations defined by their expression of DX5/CD49a or CXCR6 revealed marginally increased numbers of the trafficking DX5^+^CD49a^−^ NK cell subset already early after administration of αGalCerMPEG (Fig. S7). Liver-resident DX5^−^CD49a^+^ or CXCR6^+^ NK cells showed a transient decrease 24 h after administration followed by increased numbers as compared to untreated controls after 72 h. These data show that the administration of αGalCerMPEG impacts all NK cell populations found in the liver. In order to obtain a comprehensive insight into αGalCerMPEG-mediated modulation of all NK cells residing in the liver at the time of the analysis, the data depicted henceforward are focused on all CD3^−^NKp46^+^ NK cells.

To address whether activated iNKT cells impact the differentiation of NK cells in the liver, surface expression of CD27 and Mac-1 on CD3^−^NKp46^+^ NK cells isolated from the liver was investigated (Fig. 2B). Activation of iNKT cells by αGalCerMPEG resulted in a significantly enhanced frequency of CD27^high^ Mac-1^high^ NK cells as compared to untreated controls (Fig. 2C). This increase was accompanied by a significantly decreased frequency of CD27^low^ Mac-1^high^ NK cells. The analysis of the absolute NK cell number was in agreement with the observed increased frequency of the double positive NK cell subset (Fig. 2C). This observation was independent of the NK cell education status (Fig. S6B). These findings indicate an overall increase of functional mature CD27^high^ Mac-1^high^ NK cells in the liver. The induced modulation of the NK cell differentiation status was strictly dependent on the activation of iNKT cells, as no major αGalCerMPEG-induced changes were observed in NKT cell-deficient Jα281^−/−^ mice (Fig. 2D). This is in agreement with our recent findings of an iNKT cell-mediated generation of highly mature splenic NK cell subsets^41^. Further, our observation of an initially decreased frequency of splenic NK cells linked to the simultaneous increased frequency of NK cells in the liver hints at a dynamic homing process of splenic NK cells into the liver induced by αGalCerMPEG-stimulated iNKT cells.

### Activated iNKT cells enhance the proliferative and migratory capacity of functional mature NK cells

**Figure 3:**
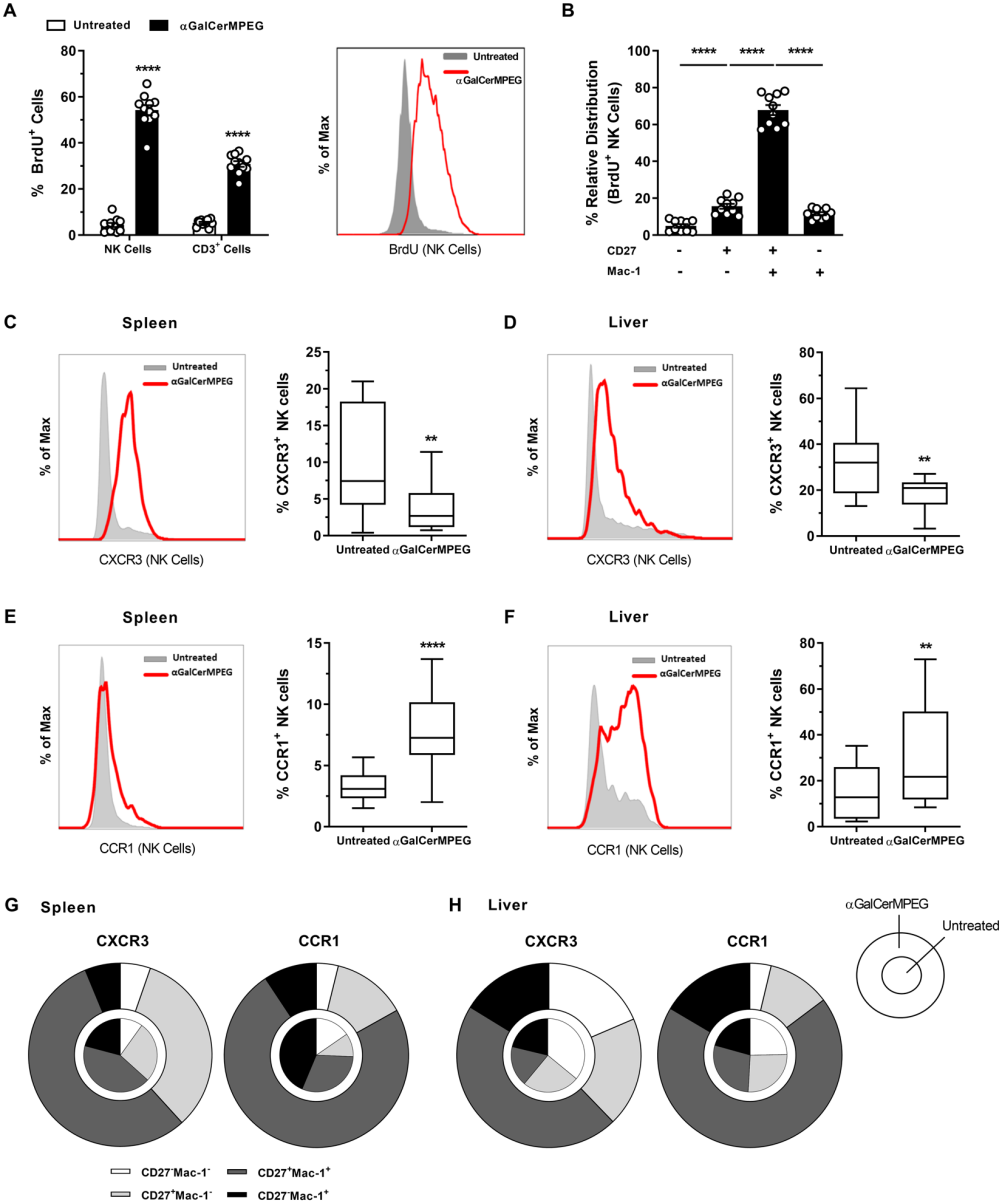
iNKT cell-induced NK cell proliferation and enhanced migratory capacity. Liver NK cells (NKp46^+^CD3^−^) and CD3^+^ cells were analyzed 72 h after s.c. injection of a single dose of αGalCerMPEG (10 µg) followed by daily intraperitoneal (i.p.) treatment with BrdU (1 mg) and subsequent incorporation analysis by flow cytometry. (**A**) Frequencies of BrdU^+^ NK and CD3^+^ cells (n = 11 mice). Histograms display a representative staining for BrdU incorporation by NK and CD3^+^ cells. (**B**) Relative distribution of BrdU^+^ NK cells (n = 10 mice). (**C**) Expression of CXCR3 by splenic and (**D**) liver NK cells (n = 14 mice). (**E**) Expression of CCR1 by splenic and (**F**) liver NK cells (n = 13–18 mice). Columns represent the mean ± SEM and circles indicate single values. Boxes represent the interquartile range, horizontal lines show the mean value and whiskers display the overall range of the data. Histograms depict the expression of CXCR3 and CCR1 by NK cells isolated from untreated (grey filled) and treated (red line) mice. (**G**,**H**) Relative distribution of splenic and liver-derived CXCR3- or CCR1-expressing NK cells displayed as “parts of the whole” (n = 13 mice, inner circle = steady state, outer ring = treatment). Pooled values from three independent experiments are shown. Asterisks denote significant values as calculated by Two-way (**A**) or One-way ANOVA (**B**) or unpaired, two-tailed Student’s t-test (**C**–**F**) as compared to untreated controls. ****p ≤ 0.0001; **p ≤ 0.01.

The observed accumulation of NK cells in the liver early after treatment might be due to changes in the proliferative or migratory capacity of NK cells mediated by activated iNKT cells. To address whether the administration of αGalCerMPEG influences the proliferative potential of NK cells in the liver, the *in vivo* incorporation of bromodeoxyuridine (BrdU) was assessed. NK cells, analyzed 72 h after iNKT cell activation, displayed significantly higher amounts of incorporated BrdU as compared to untreated controls. The αGalCerMPEG-induced proliferation was preferentially detected in the NK cell population (55%) rather than in the CD3^+^ lymphocyte population (30%) (Fig. 3A). Dissecting the NK cells based on their differentiation status explicitly depicted CD27^high^ Mac-1^high^ NK cells as having the highest proliferative capacity (Fig. 3B). This indicates that the elevated frequencies of CD27^high^ Mac-1^high^ NK cells detected following iNKT cell activation are at least in part the result of enhanced proliferation.

However, the recently described decrease in splenic NK cells following αGalCerMPEG stimulation combined with the observed enhanced NK cell counts and frequencies detected in the liver hint towards an increase in NK cell migration^41^. Thus, to address whether activated iNKT cells influence the migratory capacity of NK cells, the expression density and frequency of the spleen exit marker CXCR3 and the liver entry marker CCR1 were investigated. A decreased frequency of CXCR3^+^ NK cells was observed in both organs 72 h after αGalCerMPEG stimulation. However, splenic and liver NK cells displayed a strong increase in expression density of CXCR3 and CCR1 (Fig. 3C–F). The analysis of the NK cell differentiation status within CXCR3^+^ and CCR1^+^ NK cells revealed differential and organ-specific expression of both migration markers. Following iNKT cell activation, higher frequencies of the CD27^high^ Mac-1^high^ subset were detected within the CXCR3^+^ and CCR1^+^ splenic NK cells as compared to untreated controls, although the observed increase was much more prominent for CCR1^+^ NK cells (Fig. 3G). Activation of iNKT cells further resulted in increased frequencies of CD27^high^ Mac-1^high^ cells within the CXCR3^+^ and CCR1^+^ liver NK cell populations (Fig. 3H). These observations suggest a preferential migration of highly functional splenic NK cells into the liver upon iNKT cell activation.

### NK cell migration into the liver correlates with increased IP-10 expression by hepatocytes

The expression of CD1d by hepatocytes and their contribution to hepatic iNKT cell activation has already been described^45–47^. To investigate one potential mechanism by which hepatocytes might contribute to αGalCerMPEG-induced homing of splenic NK cells towards the liver, the expression of IP-10, an important ligand for CXCR3, was assessed 12 h after administration of αGalCerMPEG.

**Figure 4:**
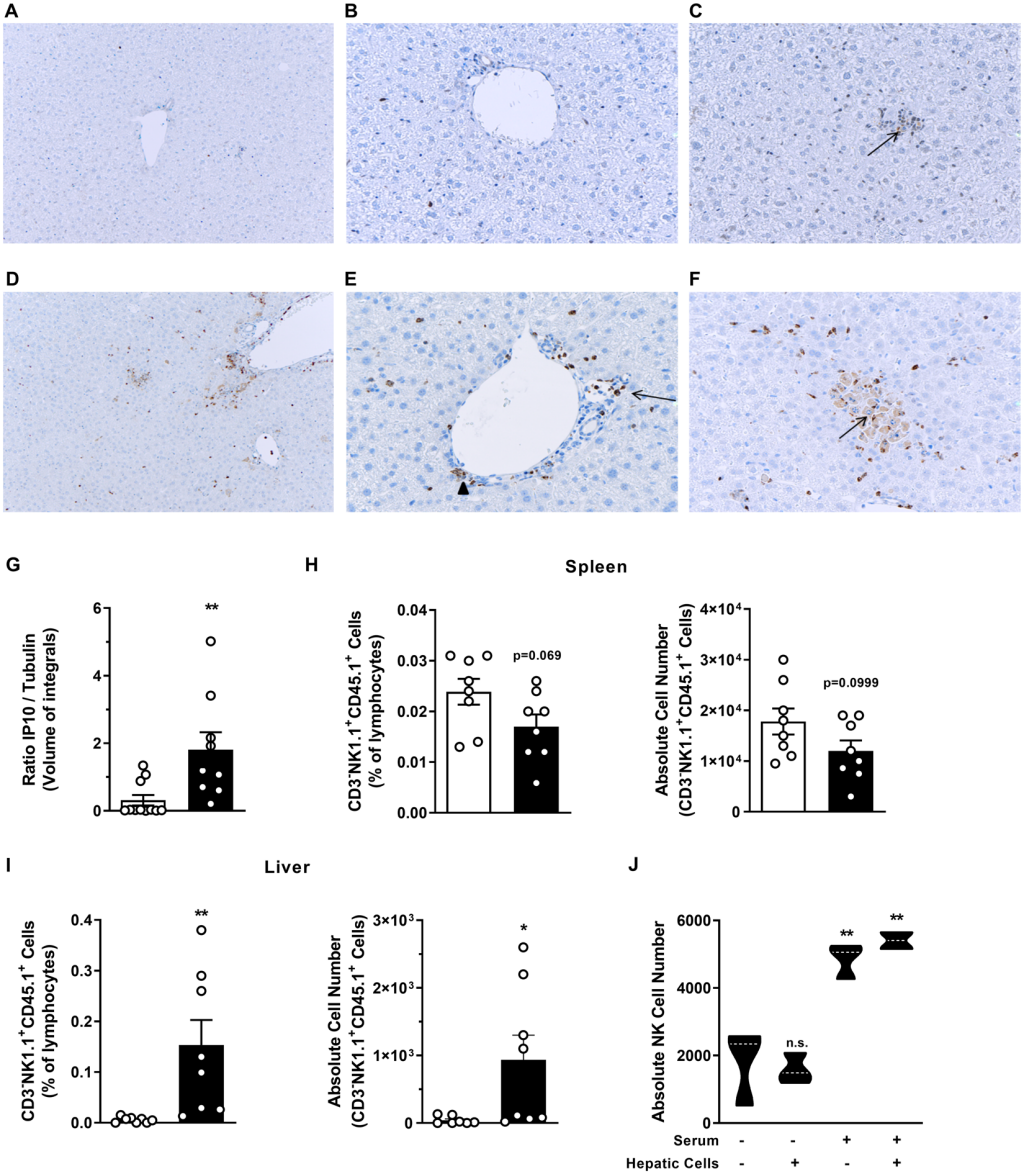
Increased hepatic IP-10 expression and liver homing of NK cells following iNKT cell activation. Livers of wt mice were excised 12 h after s.c. administration of a single dose of αGalCerMPEG (10 µg) and prepared for histopathological or Western blot analysis. (**A**–**F**) histopathological analysis of IP-10 expression (brown) in the liver of untreated (**A**–**C**) and treated (**D**–**F**) mice at an original magnification of x10 (**A**,**D**) and x20 (**B**,**C** and **E**,**F**). (**G**) Western blot analysis of monomeric IP-10 expression in hepatocytes isolated from untreated and αGalCerMPEG- administered mice (n = 9–11 mice, shown is one out of at least three independent experiments). The data are presented as the ratio of the volume of integrals of the signals detected for IP-10 and α-tubulin. For the *in vivo* migration studies, CD45.1^+^ NK cells were adoptively transferred intravenously (i.v.) into wt mice. Recipient mice were administered s.c. a single dose of αGalCerMPEG (10 µg) 24 h later and lymphocytes derived from spleen and liver were analyzed 72 h after treatment. Frequencies and absolute numbers of CD3^−^NK1.1^+^CD45.1^+^ cells (out of 1 × 10^6^ total cells) derived from (**H**) the spleen and (**I**) the liver of untreated (white) and treated (black) mice. Shown are the compiled data derived from two independent experiments (n = 7–8 mice). (**J**) The mechanism of NK cell migration was assessed using a trans-well system. Hepatocytes, serum and NK cells were obtained from untreated (−) or treated (+) mice 12 h after administration of a single dose of αGalCerMPEG (s.c., 10 µg). Absolute numbers of CFSE^+^ NK cells (out of 1 × 10^6^ total cells) were assessed after 2 h incubation at 37 °C (n = 3 mice, shown is one out of two independent experiments). Columns represent the mean ± SEM and circles indicate single values. Violin plots represent the interquartile range, horizontal lines show the mean value and the width displays the distribution of data points. Asterisks denote significant values as calculated by unpaired, two-tailed Student’s t-test as compared to untreated control. **p ≤ 0.01, *p ≤ 0.05, n.s. = not significant.

The histopathological analysis revealed that administration of αGalCerMPEG resulted in elevated numbers of IP-10 positive cells in the liver as compared to untreated controls (Fig. 4A–F). Single IP-10 positive cells (arrow) detected in untreated samples are localized in a small area of cells (Fig. A-C). The IP-10 positive cells observed in αGalCerMPEG-treated mice were mainly found in close proximity to vessels (arrowhead) and localized to endothelial cells (arrow; Fig. 4E), whereas the few IP-10 positive cells found in untreated samples were distributed within the hepatic tissue (Fig. 4B). Formation of IP-10 clusters was detected in hepatocytes of treated but not untreated livers (arrow; Fig. 4F,C). Western blot analysis was performed to investigate whether the increased number of IP-10^+^ cells detected by histopathology correlates with enhanced hepatic protein levels of monomeric IP-10 12 h after iNKT cell activation. Hepatocytes displayed increased protein levels of monomeric IP-10 as compared to untreated controls (Figs 4G and S8). These findings support the hypothesis of iNKT cell-mediated changes in the migratory capacity of NK cells that in turn might contribute to the observed alterations of NK cell numbers and NK cell subset distribution in the liver. Besides the investigated hepatic cell populations, the liver harbors a high amount of myeloid cells, including Kupffer cells^48,49^. The administration of αGalCerMPEG resulted in enhanced expression of CD80 by cDC1s and cDC2s (Fig. S9). Furthermore, enhanced frequencies of classical macrophages and decreased frequencies of Kupffer cells, pre-DCs and pDCs were observed as early as 12 h post treatment. The tSNE analysis of CD45^+^ hepatic cells showed a strong modulation of cell surface markers associated with various myeloid cell types upon αGalCerMPEG stimulation (Fig. S10).

The impact of αGalCerMPEG on the migratory behavior of NK cells was further assessed by an *in vivo* migration assay. For this, CD45.1^+^ NK cells were adoptively transferred into CD45.2^+^ wt mice, which were subsequently treated s.c. with αGalCerMPEG. After 72 h, no significant changes were observed with regard to the frequencies or absolute numbers of CD3^−^NK1.1^+^CD45.1^+^ NK cells in the spleen (Fig. 4H). However, significantly higher frequencies and absolute cell numbers of CD3^−^NK1.1^+^CD45.1^+^ NK cells were detected in the liver upon treatment with αGalCerMPEG as compared to untreated controls (Fig. 4I) suggesting an iNKT cell-induced homing of spleen-derived NK cells into the liver. Additionally, a trans-well migration system was applied to address whether ligands expressed on the surface of hepatocytes or soluble factors, derived from any cell population, are required for the proposed αGalCerMPEG-induced NK cell homing to the liver. Hepatocytes and serum derived from untreated or αGalCerMPEG-treated mice were used to analyze the migratory behavior of NK cells. Absolute NK cell numbers counted in the lower chamber after 2 h of incubation were used as a correlate for migration *in vivo*. The wells containing serum from untreated mice showed a basal level of migration. The highest number of NK cells was detected in wells containing serum from mice administered αGalCerMPEG (Fig. 4J). Hepatocytes derived from mice treated with the iNKT cell agonist did not induce additive changes with respect to NK cell counts. Thus, in this experimental *in vitro* setting, soluble factors rather than receptors expressed on the hepatocyte surface seem to be required for boosting splenic NK cell migration towards the liver following iNKT cell stimulation. These combined data foster the hypothesis that iNKT cell activation can modulate the migratory properties of NK cells thereby directing the homing of splenic NK cells into the liver.

### Activated iNKT cells affect Eomes-expressing NK cells in the liver

**Figure 5:**
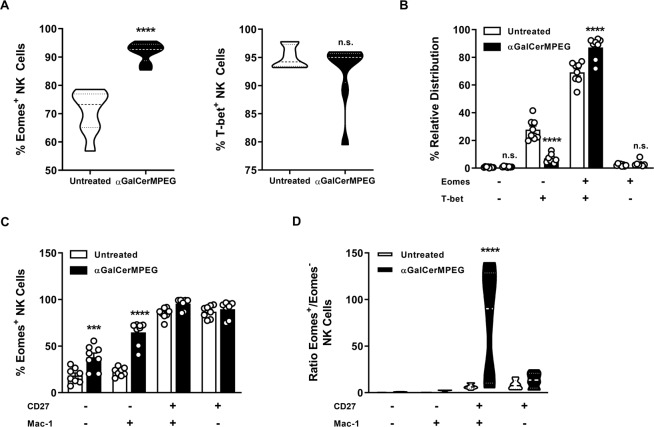
Impact of αGalCerMPEG-stimulated iNKT cells on Eomes-expressing NK cells. The expression of Eomes and T-bet by hepatic NK cells was detected 72 h after s.c. administration of a single dose of αGalCerMPEG (10 µg). (**A**) Frequencies of Eomes and T-bet expressing NK cells. (**B**) Relative distribution of NK cell subsets defined by the expression of Eomes and T-bet. (**C**) Relative distribution of CD27- and Mac-1-expressing subsets within the population of Eomes^+^ NK cells. (**D**) Ratio of Eomes^+^ and Eomes^−^ NK cells within the subsets defined by the expression of CD27 and Mac-1 (n = 8 mice, data compiled from two independent experiments). Violin plots represent the interquartile range, horizontal lines show the mean value and the width displays the distribution of data points. Columns represent the mean ± SEM and circles indicate single values. Asterisks denote significant values as calculated by One-way ANOVA (**A**), unpaired, two-tailed Student’s t-test (**B**) and Two-way ANOVA (**C**) as compared to untreated controls. ****p ≤ 0.0001; ***p ≤ 0.001; n.s. = not significant.

The transcription factors Eomes and T-bet represent important checkpoints for NK cell development. Thus, changes in their expression profile following αGalCerMPEG stimulation were investigated. Furthermore, under steady state conditions, splenic and liver-resident NK cells were reported to differ in their expression of Eomes, being significantly lower in liver-resident NK cells, whereas T-bet is equally expressed in both organs^50^. In line with this, tissue-resident NK cells contain a higher proportion of Eomes^−^T-bet^+^ cells as compared to conventional, circulating ones^22,50–52^. Following iNKT cell activation, increased frequencies of liver NK cells expressing Eomes were observed, but no significant changes were detected regarding the frequencies of T-bet-expressing NK cells (Fig. 5A). The relative distribution of Eomes- and T-bet-expressing NK cells further revealed that administration of αGalCerMPEG resulted in significantly reduced frequencies of Eomes^−^T-bet^+^ cells and significantly enhanced frequencies of Eomes^+^T-bet^+^ cells (Fig. 5B). The analysis of Eomes expression within the differentially mature NK cell subsets revealed significantly enhanced frequencies of Eomes^+^CD27^−^Mac-1^−^ as well as Eomes^+^CD27^+^Mac-1^−^ NK cells and marginally elevated frequencies of Eomes^+^CD27^+^Mac-1^+^ NK cells in the liver (Fig. 5C). Assessing the ratio of Eomes^+^ and Eomes^−^ NK cells within the subsets defined by CD27 and Mac-1 expression, higher frequencies of Eomes^+^ cells were detected in all subsets. Furthermore, significant differences were observed within the population of CD27^+^Mac-1^+^ NK cells comparing untreated and αGalCerMPEG-treated groups (Fig. 5D). These findings show that the activation of iNKT cells induces a shift in the distribution of Eomes-expressing NK cells in the liver, which might, to some extent be due to migration from the spleen into the liver.

### The generation of highly mature NK cells results in improved antiviral activity in the liver independent of the education status

**Figure 6:**
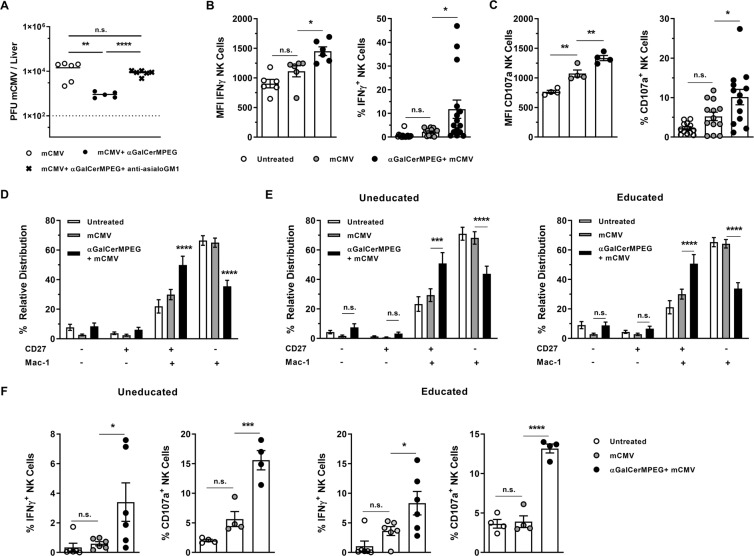
Improved antiviral activity is mediated by highly active CD27^high^ Mac-1^high^ uneducated and educated NK cells in mCMV-infected mice following iNKT cell activation. Wt mice were administered a single dose of αGalCerMPEG (10 µg) 24 h prior to infection, and livers were isolated for the assessment of the viral load by plaque assay 72 h after infection. (**A**) Viral load in the livers of infected but untreated mice (open circle), infected and treated mice (filled circle) and infected and treated mice depleted of NK cells (cross). Shown is one out of two independent experiments. For NK cell depletion, mice were treated with 80 µg/mouse of anti-asialoGM1 antibody 24 h prior to administration of αGalCerMPEG and on the day of infection. For the differentiation and functional analysis, wt mice were administered a single dose of αGalCerMPEG (10 µg). Mice were infected with mCMV 72 h later. NK cells (NKp46^+^CD3^−^) were analyzed for the expression of CD27 and Mac-1 as well as IFNγ and CD107a 24 h after infection. Frequencies and MFI of liver-derived NK cells (**B**) secreting IFNγ and (**C**) expressing CD107a (n = 8–12 mice). (**D**) Relative distribution of liver-derived NK cells (n = 15 mice). (**E**) Relative distribution of uneducated (Ly49C/I^−^ and NKG2A^−^) and educated (Ly49C/I^+^ or NKG2A^+^) NK cells (n = 15 mice). (**F**) Frequencies of uneducated and educated NK cells secreting IFNγ and expressing CD107a (n = 4–6 mice, one out of two independent experiments). MFI display representative data from one out of two or more independent experiments; frequencies show data pooled from at least two independent experiments. Columns represent the mean ± SEM and circles indicate single values. Asterisks denote significant values as calculated by One or Two-way ANOVA as compared to untreated but infected controls. ****p ≤ 0.0001; ***p ≤ 0.001; **p ≤ 0.01; *p ≤ 0.05; n.s. = not significant.

The putative beneficial antiviral effects resulting from iNKT cell-mediated generation of functional mature liver NK cells was investigated by exploiting a mCMV experimental infection model. Administration of αGalCerMPEG prior to mCMV infection resulted in reduced hepatic viral loads (Fig. 6A). The dependency on NK cells was confirmed by the fact that anti-asialoGM1-induced NK cell depletion reversed the antiviral effect, as evidenced by higher viral loads in the liver as compared to NK cell -competent treated mice (Fig. 6A).

Functional analysis of NK cells revealed that iNKT cell activation 72 h prior to mCMV infection yielded significantly increased frequencies of IFNγ-secreting and degranulating NK cells when compared to both infected but untreated and uninfected controls (Fig. 6B,C). These findings suggest that the beneficial role of iNKT cell-mediated enhanced NK cell functionality in the fight against hepatic mCMV infection can be attributed to increased IFNγ secretion and cytotoxic capacity. The improved NK cell-mediated clearance of mCMV is further supported by a significantly increased frequency of highly mature CD27^high^ Mac-1^high^ liver NK cells in mice treated with αGalCerMPEG prior to infection, as compared to both untreated infected and uninfected controls (Fig. 6D).

In the absence of αGalCerMPEG, mCMV infection did not induce changes in the differentiation status of liver NK cells as compared to untreated controls. Interestingly, the dissection of the education status showed that both uneducated and educated NK cells displayed increased frequencies of the highly functional CD27^high^ Mac-1^high^ NK cell subset in mice treated with αGalCerMPEG prior to infection. Infection with mCMV alone did not lead to significant alterations of the NK cell differentiation status within uneducated or educated NK cell populations as compared to untreated controls (Fig. 6E). To further elucidate the relevance of the education status, the functionality of uneducated as well as educated liver NK cells was assessed upon iNKT cell activation and in the course of mCMV infection. Interestingly, the frequencies of IFNγ-secreting and degranulating uneducated and educated NK cells was significantly increased upon iNKT cell activation prior to infection, as compared to both untreated but infected and untreated uninfected mice (Fig. 6F). In conclusion, in the mCMV infection model, αGalCerMPEG-mediated differentiation of liver NK cells results in enhanced antiviral activity, regardless of the education status. These findings demonstrate the beneficial effect of iNKT cell activation on crucial NK cell features rendering the iNKT-NK cell axis as a promising target for the establishment of immune interventions against liver diseases.

## Discussion

Hepatic NK cells represent a crucial innate lymphocyte population in the fight against infectious pathogens rendering them an interesting target for the establishment of immune interventions against liver diseases. The hepatic microenvironment and the high abundance of liver-resident iNKT and NK cells shape a unique environment enabling reciprocal regulation and activation^19,53,54^. Although this immune network represents a promising yet challenging target for clinical applications, a detailed understanding of liver NK cell modulation as well as the identification of compounds acting on distinct NK cell features is required. We recently demonstrated that αGalCerMPEG-activated iNKT cells affect splenic NK cell differentiation and stimulate mainly educated NK cells, thereby highlighting the potential of the iNKT-NK cell axis as a target for immune interventions. The hepatic environment, however, strongly differs from any other organ with respect to the harbored cell types as well as the immune tolerant status. In the present study, we addressed whether iNKT cell stimulation can beneficially modulate liver NK cells with a special focus on their functionality, differentiation status and migratory capacities. Our findings demonstrate that αGalCerMPEG-activated iNKT cells are potent mediators of liver NK cell stimulation. Along with the increased frequencies of cytokine-secreting and degranulating NK cells located in the liver, enhanced frequencies and absolute cell numbers of the highly mature CD27^high^ Mac-1^high^ NK cell subset were observed. These observations are in line with the described differentiation stages of NK cells, which define the CD27^high^ Mac-1^high^ subset as the most functional one^10^. It needs to be considered that next to conventional NK cells (CD3^−^NKp46^+^), the liver harbors permanently residing NK cells commonly defined by the expression of CD49a and the lack of DX5 expression. Another liver-residing NK cell population is defined by the expression of CXCR6, which is often referred to antigen-specific innate memory responses and liver-retention^28,42,55^. The observed impact of αGalCerMPEG on DX5^−^CD49a^+^ and CXCR6^+^ NK cells show that not only conventional but also liver-resident NK cells are affected by the treatment. This is in line with their functional properties described in the course of hepatic infections^56^. The activation of different NK cell populations present in the liver might be beneficial for the maintenance of a balanced and liver-adapted immunity. Due to the fact that lymphocyte recruitment and migration are important immune features, liver-resident as well as transiently resident NK cells, including circulating and recruited ones, were addressed by assessing CD3^−^NKp46^+^ cells.

Although the underlying mechanisms leading to the modulation of liver-resident NK cells have not been completely elucidated, iNKT cell-derived cytokines are most probably the main driving force. We reported earlier that αGalCerMPEG treatment leads to increased serum levels of various iNKT-, NK- and antigen presenting cell-related cytokines^41^. Here, IL-12 has been linked to enhanced NK cell activity and degranulation^57,58^. *In vitro* blocking experiments suggested that IL-12 plays a major role, whereas IFNγ and IL-2 are marginally relevant for the αGalCerMPEG-mediated induction of NK cell cytotoxicity (data not shown). This is in agreement with reports describing IFNγ as an important mediator for the activation of the death receptor pathway, but not degranulation in NK cells^59,60^.

The impact of activated iNKT cells on NK cell migration has not been addressed so far. The results gained from our *in vivo* migration assay revealed that the administration of αGalCerMPEG induces the homing of conventional CD3^−^NKp46^+^ NK cells to the liver. Changes in serum soluble factors induced by αGalCerMPEG appear to support the migratory capacity of splenic NK cells, thereby resulting in enhanced liver homing. This effect might be facilitated by a chemokine gradient directing CXCR3-expressing NK cells to the liver. The importance of CXCR3 and its ligands for the recruitment of lymphocytes to the liver is well documented^61–63^. Furthermore, the migration of DCs was shown to be jointly directed by chemokine fields and soluble chemokine gradients^64^. In the context of NK cells, a positive gradient of MCP-1 was reported to induce maximal migration of IL-2-activated NK cells *in vitro*^65–67^. Thus, the detected enhanced serum IP-10 levels following iNKT cell activation might contribute to the observed NK cell migration. This is supported by studies showing that DC-derived IP-10 can induce NK cell influx to the peritoneal cavity upon (i.p.) injection of a cell vaccine or CpG-activated plasmacytoid DCs^68^. The importance of CXCR3- and IP-10-dependent NK cell recruitment is further confirmed by studies using IFNγ and CXCR3 knockout mice. Here, decreased survival rates upon tumor challenge were observed due to a reduced NK cell accumulation^69,70^. The hypothesis of an iNKT cell-mediated migration of splenic NK cells towards the liver is thus further underpinned by an increased expression density of CXCR3. In this context, CXCR3-dependent NK cell infiltration was shown to be important for the lysis of tumor cells^62,68,70,71^. Administration of a different iNKT cell agonist (αGal-C18-Cer) was also observed to induce CXCR3-dependent recruitment of regulatory T cells to the liver^72^. In contrast to the expression density, a decreased frequency of CXCR3^+^ NK cells was observed in the spleen and the liver upon iNKT cell stimulation. This might arise due to receptor degradation upon binding of its ligand as described earlier^73^. The expression of the CXCR3 ligand IP-10 is strongly associated with the induction of an efficient immune response by mediating lymphocyte recruitment and activation, as shown for various hepatic infections^74–78^. The observed increased hepatic IP-10 expression thus might indicate that iNKT cell activation drives NK cell migration in a dual fashion, by the induction of both, CXCR3 expression by NK cells and hepatic IP-10 expression. Previous publications hint that oligomeric forms of IP-10 might also contribute to the described observations. In this context, an equilibrium of monomers and dimers was observed in free solution and oligomerization of secreted monomeric IP-10 was shown to be necessary for endothelial cell presentation and its activity *in vivo*^79,80^. More recently, also a tetrameric form of IP-10 was described^81^. The performed western blot analysis might support the oligomerization of IP-10 and this should be considered for further studies. However, IP-10 is described not to be detectable in normal liver tissue and strong reducing conditions were applied^82^. Therefore, the shown analysis was focused on the monomeric form. The histopathological and western blot analyses revealed that hepatocytes are most likely the main source of IP-10. This is supported by a report describing enhanced IP-10 mRNA levels in hepatocytes located in inflammatory areas as well as the detection of IP-10 in the cytoplasm of hepatocytes, but not any other cells, in liver biopsy tissue chronic hepatitis C patients^75,83^. Nevertheless, other hepatic IP-10-producing cell types, such as endothelial cells, neutrophils, macrophages, monocytes or Kupffer cells might support the establishment of an IP-10 gradient and should be considered in future studies^76,84–87^. This is supported by the finding that administration of αGalCerMPEG impacts various hepatic DC populations as well as classical macrophages and Kupffer cells. These cells of the myeloid lineage are crucial for the suppression of inflammation and infections in the liver as well as for tissue regeneration but also for the progression of liver diseases^88,89^. Future studies are needed to assess the impact of different hepatic cell populations and their value for clinical approaches. Dissection of the differentiation status of NK cells showed that mainly CD27^high^ NK cells displayed an enhanced expression of CXCR3. Previous studies reported an increased expression of CXCR3 under steady state within the splenic CD27^high^ Mac-1^high^ subset as compared to CD27^low^ Mac-1^high^ cells^10^. Here we demonstrate for the first time that iNKT cell stimulation results in improved NK cell migration of both CD27^high^ subsets. This is further supported by the finding that upon iNKT cell activation splenic CD27^high^ Mac-1^low^ NK cells showed increased expression of CCR1, a marker connected to immune cell homing to the liver in inflammatory settings^90,91^. The sequential requirement for CXCR3 and CCR1 for the mobilization of splenic NK cells and their subsequent accumulation in the liver was demonstrated in a murine Con A-induced hepatitis model. Therefore, CCR1 might contribute critically to the liver entrance once the activated NK cells reach the liver.

Remarkably, NK cells found in the liver displayed a high expression of the transcription factor Eomes after iNKT cell stimulation. In more detail, administration of the iNKT cell stimulus led to a parallel decrease of Eomes^−^T-bet^+^ and increase of Eomes^+^T-bet^+^ NK cells. In general, conventional circulating DX5^+^ NK cells contain a higher proportion of Eomes^+^T-bet^+^ cells as compared to liver-resident DX5^−^ NK cell populations^22,51,52,92^. Thus, the shift in Eomes- and T-bet-expressing NK cells might hint at an influx of circulating NK cells to the liver induced by iNKT cell stimulation. This is consistent with the high ratio of Eomes^+^/Eomes^−^ NK cells within the CD27^+^Mac-1^+^ NK cell subset harboring high migratory capacity. Furthermore, CD27^−^Mac-1^−^ NK cells are described to harbor a high capacity for homeostatic proliferation and thus enhanced frequencies of Eomes^+^CD27^−^Mac-1^−^ NK cells hint at a higher proliferative capacity of Eomes-expressing NK cells in the liver upon αGalCerMPEG administration^12^. However, a stress-induced expression of Eomes by hepatic NK cells can also not be excluded. Furthermore, Eomes expression by human CD56^bright^ NK cells was shown to be inducible upon stimulation with IL-12 + IL-15 or IL-12 + IL-18^22^. Stimulation with αGalCerMPEG might thus likewise impact Eomes expression.

The maintenance of hepatic immune tolerance is a major issue with regard to immune modulation, in order to prevent undesired immune activation. In close interaction with regulatory T cells, NK cells constitute one essential cell population supporting tolerance by the direct inhibition of DCs and the secretion of regulatory cytokines. In this regard, IL-10 secreted by NK and NKT cells, amongst other cell populations, was described to be essential to retain immune tolerance^93,94^. Our findings revealed that iNKT cell activation did not affect serum levels of IL-10, thereby suggesting that the tolerant status is maintained (Fig. S11). This is in consistence with our observations that αGalCerMPEG administration does not induce changes in the level of IL-10 production by NKT cells (data not shown). The finding that αGalCerMPEG mainly impacts the functionality of educated NK cells renders the disturbance of the immune balance by activation of NK cells rather unlikely. However, the meaning of the education status with respect to the maintenance of hepatic immune tolerance still needs to be elucidated to advance the use of hepatic NK cells as targets for immune interventions without causing dysregulated immune reactions.

The interaction of αGalCer (MPEG)-activated iNKT cells with NK cells is well demonstrated^40,41,95,96^. However, it needs to be considered that four different NKT cell subsets (NKT1, NKT2, NKT17 and NKT10) harboring different effector functions are described^43,97^. The observed high serum levels of IFNγ and IL-4 early after αGalCerMPEG administration hint towards the activation of especially the NKT1 as well as the NKT2 subsets^41,98^. The distinct analysis of the NKT cell subsets showed that NKT1 cells appear to be the main responding population. This is line with the described tissue distribution of the NKT cell populations allocating NKT1 cells mainly to the liver and the spleen whereas NKT2 and NKT17 cells are mainly found in the lung and peripheral LNs, respectively^43,99^.

Next to the different immune cell populations, hepatocytes themselves might also contribute to the αGalCerMPEG-induced effects. It has already been described that iNKT cells can become stimulated by lipid antigens presented by CD1d-expressing hepatocytes^100,101^. Furthermore, cytokines released by activated antigen-presenting cells (APCs), NKT and NK cells as well as additional bystander cell populations might contribute to hepatocyte activation and subsequently lead to the secretion of immune modulating factors^102,103^.

The administration of αGalCerMPEG prior to infection with mCMV clearly demonstrated that iNKT cell-mediated NK cell activation exerts beneficial effects in supporting the clearance of viral infections. These findings are in line with observations made for the parental, non-pegylated compound αGalCer^96^. The importance of NK cells in the defense against mCMV infection was evaluated by anti-asialoGM1 antibody-induced NK cell depletion. Enhanced viral loads in NK cell-depleted as compared to NK cell-competent mice clearly prove their antiviral role upon iNKT cell activation. Yet, it needs to be considered that this NK cell depletion method can also result in a minor depletion of additional cell populations e.g. basophils and a subpopulation of CD8^+^ T cells or even directly impact the course of infection^104,105^. IFNγ release and degranulation represent crucial factors in the NK cell-mediated response against CMV^106–109^. Therefore, the αGalCerMPEG-induced secretion of IFNγ by NK cells and their enhanced killing capacity, together with the observed elevated frequencies of mature CD27^high^ Mac-1^high^ NK cells, might represent fundamental underlying mechanisms leading to an improved viral clearance. Surprisingly, in the course of mCMV infection, the functionality of NK cells following iNKT cell stimulation with αGalCerMPEG was independent of the education status. Although uneducated NK cells are considered to be hypo-responsive, previous studies have emphasized their impact in the clearance of mCMV infection^110,111^. Our findings revealed an αGalCerMPEG-induced activation restricted to educated NK cells. However, during viral infection the stimulation of iNKT cells results not only in the activation of educated but also uneducated NK cells. Thus, the observed beneficial effect during viral infection following αGalCerMPEG treatment is most probably due to the activation of both educated and uneducated NK cells by activated iNKT cells as well as the viral infection itself.

The potential of NK cells in clinical settings is already under exploitation for the treatment of cancer^112,113^. However, targeting NK cells via the iNKT cell axis to combat viral infections or for the design of advanced vaccination strategies represents a novel research area. The data presented here clearly demonstrate the potential of NK cell modulation for prophylactic or therapeutic immune interventions against infectious liver diseases and highlight the complexity of the αGalCerMPEG-induced responses. In this context, also the memory-like features of the liver-resident CD49a^+^DX5^−^ or CXCR6^+^ NK cell populations represent interesting characteristics for the described iNKT cell-mediated modulation^42,56^. Thus, the generation of memory-like NK cells by iNKT cell activation would represent a fundamental step towards the development of innovative strategies for immune interventions.

## Material and Methods

### Animals

Wild type mice (C57BL/6 (H-2b)) were purchased from Harlan Winkelmann GmbH (Borchen, Germany). Jα281^−/−^ and CD45.1 transgenic mice were bred in-house at the animal facility of the Helmholtz Centre for Infection Research, Braunschweig. All mice were held in individual ventilated cages under pathogen free conditions with food and water ad libitum. The treatment was performed in accordance with local and European Community guidelines. The described animal handling and procedures were approved by the local government in Braunschweig (Germany) under the animal permission codes 33.9-42505-04-12/0942, 33.42502-13/1305 and 33.19-42502-04-16/2280.

### αGalCerMPEG synthesis and treatment

αGalCerMPEG was synthesized from αGalCer and methyl-PEG-COOH as described earlier^40^. The final purification step was performed by silica gel chromatography and a high purity (96%) was confirmed by HPLC analysis. A single dose of αGalCerMPEG (3.4 nmol (10 µg) in 50 µl PBS) was administered subcutaneous (s.c.). Single cell preparations were prepared at the indicated time points and the cells were phenotypically and functionally analyzed.

### mCMV infection model

Mice were infected by i.p. route with the wild type mCMV (Smith strain repair, pSM3fr-MCK-2fl) 24 h or 72 h after s.c. administration of a single dose of αGalCerMPEG (10 µg). C57BL/6 mice were infected with 1 × 10^6^ PFU mCMV. The virus preparation was carried out as recently described^114^. Briefly, the virus was grown in mouse embryo fibroblasts and purified by sucrose cushion density centrifugation. The hepatic viral load was determined by plaque assay at the indicated time points. NK cell depletion was performed by i.p. administration of 80 µg anti-asialoGM1 24 h prior to αGalCerMPEG treatment and at the day of infection.

### Preparation of hepatic and splenic single cell suspensions

To obtain hepatic lymphocyte suspensions, livers were perfused with PBS, minced through a 100 µm cell mesh using medium containing collagenase D (0.1 U/ml)/DNase (0.1 mg/ml) and digested for 20 min at 37 °C. Subsequently, cells were centrifuged for 3 min at 300 rpm, followed by hepatic lymphocytes purification by density gradient centrifugation (Easycoll separating solution, ρ = 1.124 g/ml, Biochrom AG). Splenic single cell suspensions were prepared as described earlier^41^. Briefly, spleens were minced through a 100 µm cell mesh and the resulting single cell suspensions were subjected to erythrocyte lysis.

### Isolation of hepatocytes

The isolation of hepatocytes was performed as described^115^. Briefly, livers were perfused sequentially with perfusion buffer and collagenase D-containing digestion buffer (0.16 mg/ml) and subsequently digested in digestion buffer containing DNase (10 µg/ml) for 10 min at 37 °C. The resulting cell suspension was sequentially filtered through 100 µm and 70 µm cell meshes and centrifuged. Hepatocytes were purified by density gradient centrifugation (Ficoll-Paque PLUS, ρ = 1.078 g/ml, GE Healthcare Life Sciences).

### Isolation of hepatic cells for flow cytometry analysis

Livers were perfused with 2 ml PBS per organ, chopped in fine pieces using scissors and incubated in medium containing collagenase D (0.652 mg/ml), DNase (0.1 mg/ml) and dispase (1 mg/ml) at 37 °C for 25 min. The samples were mixed every 5 min. Subsequently, the digests were poured through a 100 µm filter and centrifuged at 300 × g for 5 min. The cells were washed and RBC lysis was performed.

### Flow cytometry analysis

To perform a flow cytometry-based phenotypic and functional analysis of NK cells, the isolated lymphocytes were incubated with FcR-block and surface staining was performed for 20 min at 4 °C in the dark in PBS. For the intracellular staining, cells were subsequently treated with Cytofix and Cytoperm (BD Bioscience, USA) according to the manufacturer’s protocol. Briefly, cells were fixed using Cytofix and the staining was carried out for 20 min at 4 °C in the dark in Cytoperm wash buffer. FACS analysis was performed by FACS LSRII and Fortessa (BD Bioscience, USA) and FlowJo (TreeStar Inc). The following antibodies were used for FACS staining: CD3 (500A2, V500, BD Horizon; 145-2C11, BUV395, BD Horizon; 17A2, BV650, BioLegend), NKp46 (29A1.4, eFluor660; eFluor450 eBioscience), DX5 (DX5, APC-Cy7, BioLegend), Mac-1 (M1/70, Pacific Blue, eBioscience; FITC, BD; BV421, BioLegend; V500, BD Horizon; BUV395, BD), CD27 (LG.7F9, PE-Cy7, eBioscience; LG.3A10, PE-Dazzle594, BioLegend), CD69 (H1.2F3, PE, BD; BV605, BioLegend), Streptavidin (BV 605, BioLegend), CXCR3 (CXCR3-173, APC, BioLegend), CCR1 (643854, PE, R&D Systems), IFNγ (XMG1.2, PE, eBioscience; BV785 or BV711, BioLegend), CD107a (1D4B, APC-Cy7 or BV421, BioLegend; eBio1D4B, FITC, eBioscience), Ly49C/I (5E6, PE, BD Pharmingen), NKG2A/C/E (20d5, FITC, BD Pharmingen), CD49a (Ha31/8, BV650, BD), TRAIL (N2B2, PE, BioLegend), Eomes (Dan11mag, A488, eBioscience), T-bet (4B10, PE-Cy7, BioLegend), CXCR6 (SA051D1, BV711, BioLegend), PLZF (9E12, PerCP-Cy5.5, BioLegend), RORγ (AFKJS-9, APC, eBioscience), NK1.1 (PK136, A700, BioLegend), CD4 (GK1.5, BUV395, BD), CXCR5 (2G8, BV421, BD), PD-1 (29 F.1A12, BV605, BioLegend), TCRβ (H57-597, BV510, BioLegend), IL-17RB (6B7, BV786, BD), IL-4 (11B11, PE-Dazzle594, BioLegend), IL-17A (eBio17B7, FITC, eBioscience), CD45 (30-F11, A700, BioLegend), CD11c (N418, APC-Fire750, BioLegend), CD103 (2E7, BV510, BioLegend), MHC cl. II (AF6-120.1, PerCP-Cy5.5, BioLegend), Langerin (eBioRMUL.2, eF660, eBioscience), F4/80 (BM8, BV650, BioLegend), PDCA1 (eBio927, eF450, 3Bioscience), Ly6C (HK1.4, BV785, BioLegend), Ly6G (1A8, PE-Dazzle594, BioLegend), CX3CR1 (SA011F11, A488, BioLegend), TLR4 (SA15-21, PE-Cy7, BioLegend), TLR9 (J15A7, PE, BD), CD80 (16-10A1, BV711, BD), CD95 (SA367H8, PE, BioLegend), LIVE/DEAD Fixable Blue Dead cell stain kit (UV excitation, Invitrogen).

### *In vivo* proliferation assay

*In vivo* NK cell proliferation was assessed 72 h after administration of a single dose of αGalCerMPEG (10 µg). For this, mice received a daily i.p. injection of 1 mg bromodeoxyuridine (BrdU). Analysis was performed by flow cytometry (BrdU Flow Kit, BD Pharmingen). Samples were analyzed by FACS LSRII (BD Bioscience, USA) and FlowJo (TreeStar Inc.).

### NK cell degranulation and IFNγ secretion

To investigate NK cell functionality, isolated hepatic lymphocytes were co-incubated with YAC-1 target cells at the E:T ratio 10:1 and in the presence of an anti-CD107a antibody. To prevent internalization of CD107a and the secretion of cytokines, monensin and brefeldin A (5 µg/ml, Sigma) were added after 1 h. Cells were then cultured for a further 5 h and subsequently stained for flow cytometry analysis.

### Western blot analysis

The quantity of IP-10-expressing hepatocytes was assessed by Western blot analysis. To this end, the isolated hepatocytes were counted and equal cell numbers were re-suspended in lysis buffer containing protease inhibitor cocktail (3 µl/ml, Sigma) and calyculin A (100 nM, Cell Signaling) followed by 10 min incubation on ice with repeated vortexing. Protein sample buffer (4x concentrated) was subsequently added (40% glycerol; 240 mM Tris; HCl pH 6.8; 8% SDS; 0.04% bromophenol blue; 7% β-mercaptoethanol) and the samples were denatured at 99 °C for 5 min. Samples were stored at −20 °C and denatured again at 99 °C for 5 min upon anew addition of 7% β-mercaptoethanol. The samples were separated using a 15% SDS-polyacrylamide gel followed by electroblotting onto a nitrocellulose membrane. Membranes were incubated first with the primary antibodies against IP-10 or α-tubulin (goat anti mouse IP-10, R&D Systems; rabbit anti mouse α-tubulin, Abcam) at 4 °C overnight and second with the respective horseradish-peroxidase-conjugated antibodies. The enzymatic reaction was detected using the ECL System (Santa Cruz Biotechnology) and the images were acquired by the ChemiDoc-it System (Bio-Rad). The relative concentration of IP-10 (ratio of volume under the integral of IP-10 and α-tubulin) was assessed using Image Lab Software (5.2.1 Bio-Rad).

### Histopathological analysis

To determine the distribution of IP-10-producing cells, liver tissue was fixed in 4% formaldehyde and embedded in paraffin. Paraffin sections were de-paraffinized in xylene and rehydrated in a graded ethanol series. For antigen retrieval, slides were placed in a pressure cooker in citrate buffer (pH 7.6). Endogenous peroxidase was blocked with 3% H_2_O_2_. Subsequently, slides were blocked with ready to use blocking solution (Zytomed Systems). For the staining of liver sections, the primary antibody anti IP-10 (bs-1502R, Bioss) and a secondary biotinylated antibody (Zytomed Systems) were used. Subsequently, streptavidin–HRP conjugate (Zytomed Systems), DAB chromogen, and substrate buffer (Zytomed Systems) were added. The slides were analyzed by light microscopy, blinded to the experimental groups.

### *In vitro* migration assay

An *in vitro* migration assay was performed to address factors mediating the migration of splenic NK cells to the liver. To this end, hepatocytes and sera derived from either naive or αGalCerMPEG treated mice (described above) were placed in the lower chamber of a 96-well trans-well plate (Corning). Sorted splenic NK cells (NK1.1 (PK136, PE, eBioscience), CD4 (RM4-5, BV421, BioLegend), CD8 (53-6.7, BV421, BioLegend), CD11c (HL3, FITC, BD), and B220 (RA3-6B2, FITC, eBioscience) isolated from untreated mice were stained with carboxyfluorescein succinimidyl ester (CFSE) and placed in the upper chamber of the trans-well system. After 2 h incubation at 37 °C, cells were collected from the lower chamber and stained for NK cells (CD3^−^ (500A2, V500, BD Horizon), NKp46^+^ (29A1.4, eFluor660, eBioscience)). For the determination of absolute cell numbers, CountBright™ Absolute Counting Beads (Thermo Fisher Scientific) were added to the samples prior to the acquisition.

### *In vivo* migration assay

The migration of NK cells *in vivo* was assessed upon adoptive transfer of CD45.1^+^ NK cells. For this purpose, splenic NK cells were isolated from CD45.1 transgenic mice using the NK Cell Isolation Kit II for the isolation of untouched NK cells (Miltenyi, Germany) according to the manufacturer’s protocol. Subsequently, 5 × 10^5^ NK cells (in 100 µl PBS per mouse) were injected via the i.v. route. 24 h after the transfer, mice were administered a single dose of αGalCerMPEG as described. Splenic and hepatic lymphocytes were analyzed 72 h after treatment by flow cytometry (CD3 (17A2, BUV395, BD Biosciences), NK1.1 (PK136, PE, eBioscience), LIVE/DEAD Fixable Blue Dead cell stain kit (UV excitation, Invitrogen), CD45.1 (A20, eF450, eBioscience)).

### Stochastic neighbor embedding (t-distributed) analysis (tSNE)

Flow cytometry data was imported into FlowJo (version 10.5.3) and following compensation, samples were randomly down-sampled using the respective plug-in to reduce the event rate to 2000 events per sample. The reduced samples were labeled, concatenated and the tSNE analysis was then performed using the respective plug-in. The resulting data were plotted with intensities for the depicted marker.

### Statistical analysis

GraphPad Prism (student’s t test, One-way or Two-way ANOVA) was used for the statistical assessment of the presented data. Values of p ≤ 0.05 were considered significant.

## Supplementary information

Supplementary Information
