# Supplementary material for: Key features and homing properties of NK cells in the liver are shaped by activated iNKT cells
Source: Sci Rep. 2019 Nov 8;9:16362. doi: 10.1038/s41598-019-52666-9 (PMC6841958; doi:10.1038/s41598-019-52666-9)
Supplement: Supplementary file 1 — Supplementary Information [file 41598_2019_52666_MOESM1_ESM.pdf]

Supplementary Information

Key features and homing properties of NK cells in the liver are shaped by activated iNKT cells

Stephanie Trittel [1], Benedict J. Chambers [2], Ulrike Heise [3], Carlos A. Guzmán*[1], Peggy Riese*[1]

* Equally contributed

Figure S1

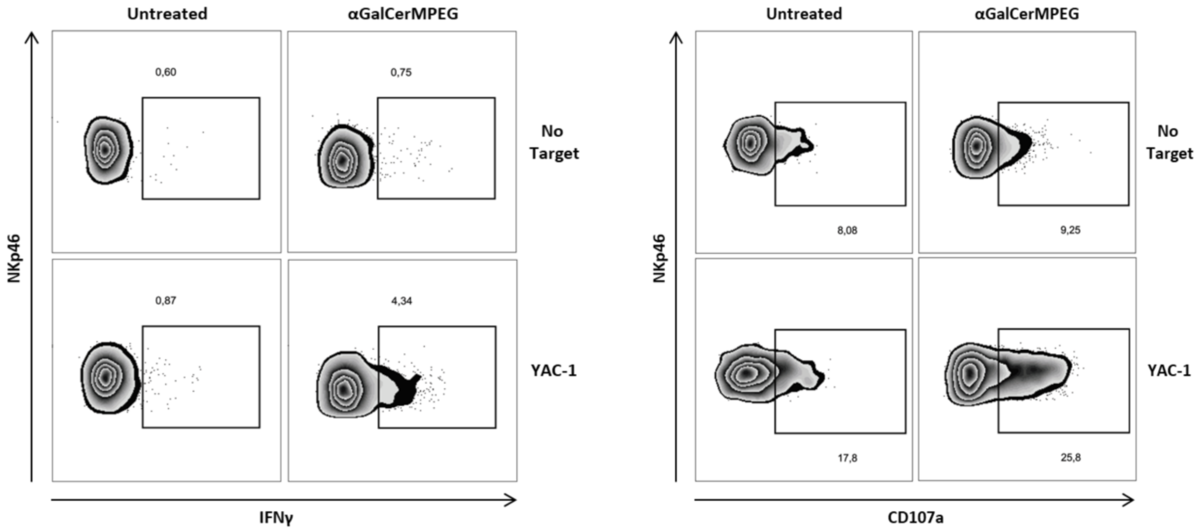

Figure S1. Representative flow cytometry plots of αGalCerMPEG-activated NK cells. Hepatic lymphocytes were isolated from wt mice 72 h after administration of a single dose of αGalCerMPEG (10 µg) and the representative dot plots show the flow cytometry gating for the expression of IFNγ and CD107a detected in cells derived from wt mice assessed after 6 h co-incubation with YAC-1 target cells.

Figure S2

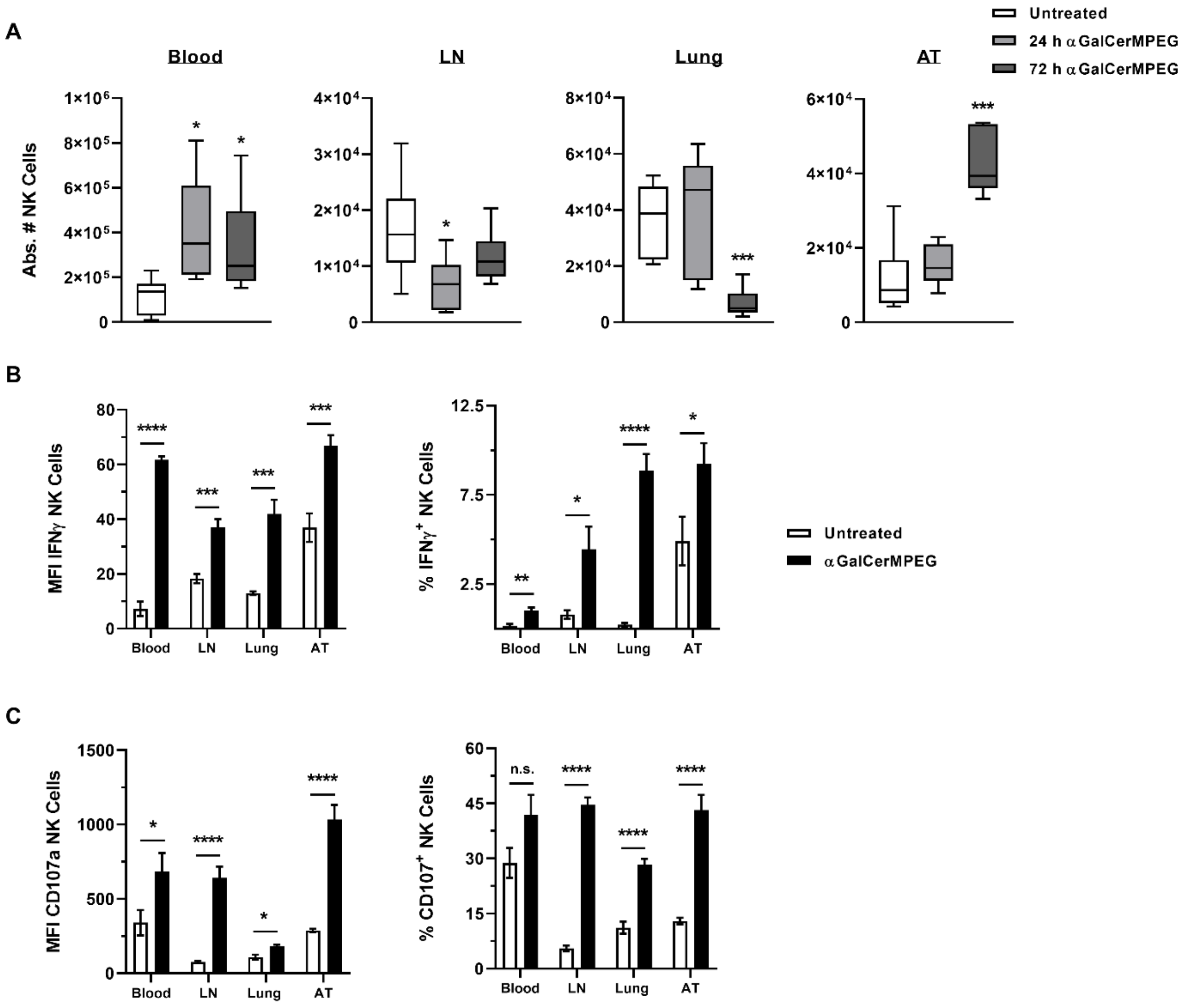

Figure S2. Functionality of NK cells derived from blood, lymph nodes, lung and adipose tissue. Lymphocytes were isolated from the blood, lymph nodes (LN), lung and adipose tissue (AT) of wt mice 72 h after administration of a single dose of αGalCerMPEG (10 µg). The expression of IFNγ and CD107a was assessed after 6 h co-incubation with YAC-1 target cells. (A) Absolute cell number of NK cells (out of $1x10^6$ total cells). MFI and frequencies of (B) IFNγ secreting and (C) CD107a expressing blood-, LN-, lung- and AT-derived NK cells. The data are derived from one experiment with n = 6 mice per group. Columns represent the mean ± SEM. Asterisks denote significant values as calculated by unpaired, two-tailed Student's t-test. **** p ≤ 0.0001;*** p ≤ 0.001; ** p ≤ 0. 01; * p ≤ 0; 05.

Figure S3

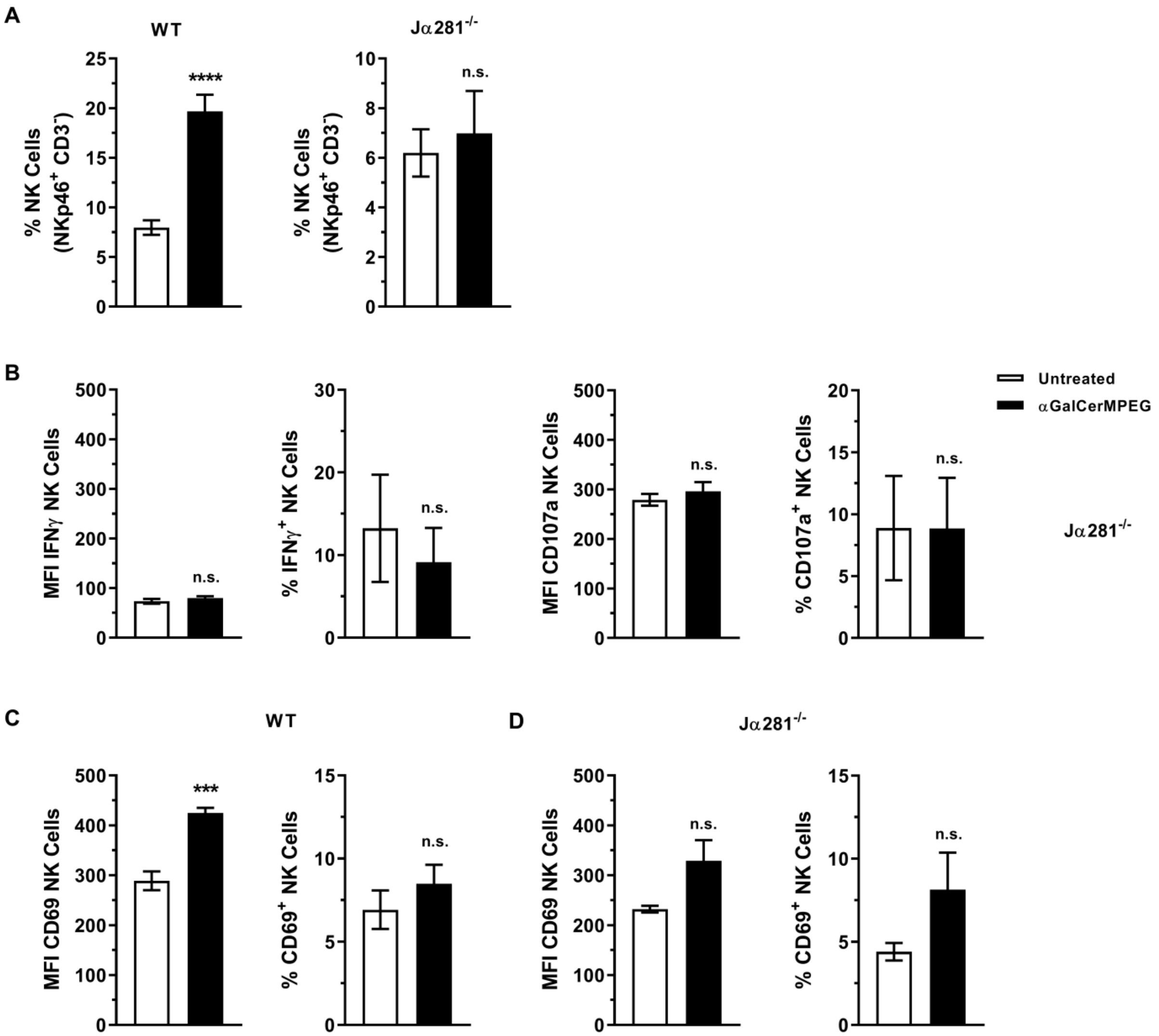

Figure S3. αGalCerMPEG-mediated NK cell activation is dependent on NKT cells. Hepatic lymphocytes were isolated from wt and Jα281$^{-/-}$ mice 72 h after administration of a single dose of αGalCerMPEG (10 µg) and the expression of IFNγ and CD107a was assessed after 6 h co-incubation with YAC-1 target cells. (A) Frequencies of NK cells (NKp46$^+$CD3$^-$) detected in hepatic lymphocytes derived from wt and Jα281$^{-/-}$ mice (n=10-15). (B) MFI and frequencies of NK cells isolated from NKT cell-deficient Jα281$^{-/-}$ mice expressing IFNγ or CD107a. (C) MFI and frequencies of NK cells isolated from wt or NKT cell-deficient Jα281$^{-/-}$ mice expressing CD69. MFI: n=4 mice, one out of at least three independent experiments. Frequencies: n=7 mice; columns represent the mean ± SEM of data pooled from two independent experiments. Asterisks denote significant values as calculated by One-way ANOVA. **** $p \leq 0.0001$; *** $p \leq 0.001$; n.s. = not significant.

Figure S4

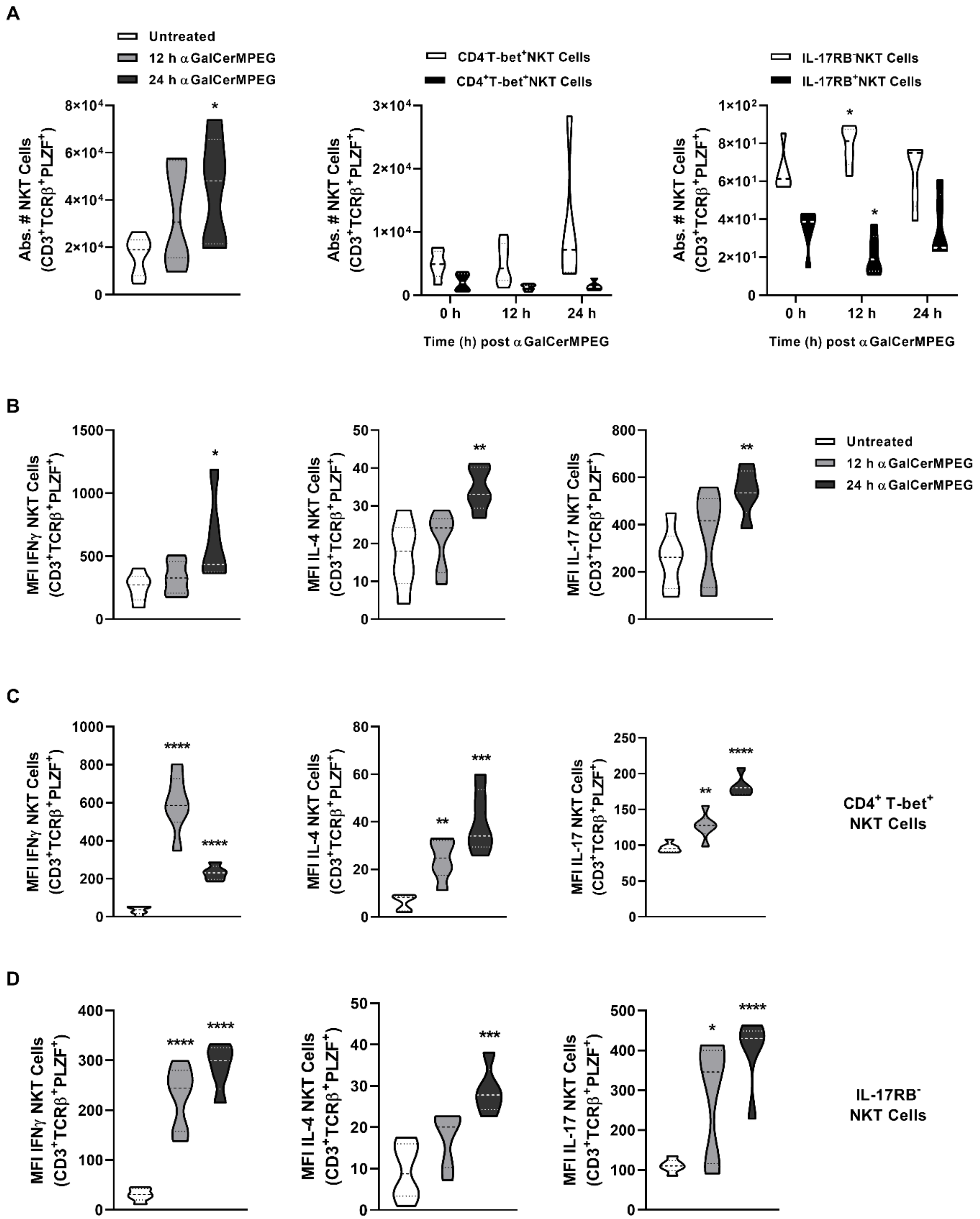

Figure S4. Phenotype and functionality of hepatic NKT cell populations. Lymphocytes were isolated from livers of mice treated with a single dose of αGalCerMPEG (10 µg) at the indicated time points. (A) Absolute cell numbers of NKT cell subsets (out of $1x10^6$ total cells). MFI of IFNγ-, IL-4- and IL-17-expressing NKT cell subsets detected as (B) $CD3^+TCR\beta^+PLZF^+$, (C) $CD3^+TCR\beta^+PLZF^+IL\text{-}4^+T\text{-}bet^+$ and (D) $CD3^+TCR\beta^+PLZF^+IL\text{-}17RB^-$. Violin plots represent the interquartile range, horizontal lines show the mean value and the width displays the distribution of data points. n=6 mice,

data derived from one experiment. Asterisks denote significant values as calculated by unpaired, two-tailed Student's t-test. **** $p \leq 0.0001$;*** $p \leq 0.001$; ** $p \leq 0.01$; * $p \leq 0; 05$.

Figure S5

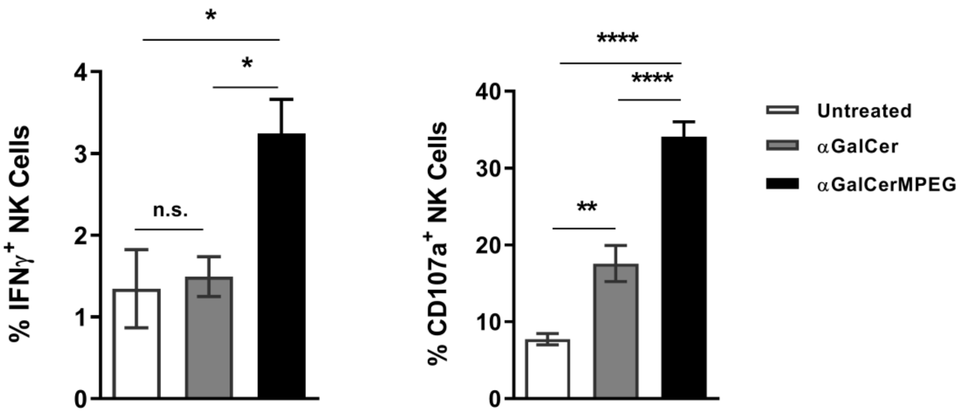

Figure S5. Comparison of NK cell activation by αGalCer and αGalCerMPEG. Hepatic lymphocytes isolated from wt mice 72 h after administration of a single dose of αGalCerMPEG or αGalCer (both 3.4 nmol) were assessed for the expression of IFNγ and CD107a after 6 h co-incubation with YAC-1 target cells (n=5 mice). Columns represent the mean ± SEM of the data. Asterisks denote significant values as calculated by One-way ANOVA. **** $p \leq 0.0001$; ** $p \leq 0.01$; * $p \leq 0.05$; n.s. = not significant.

Figure S6

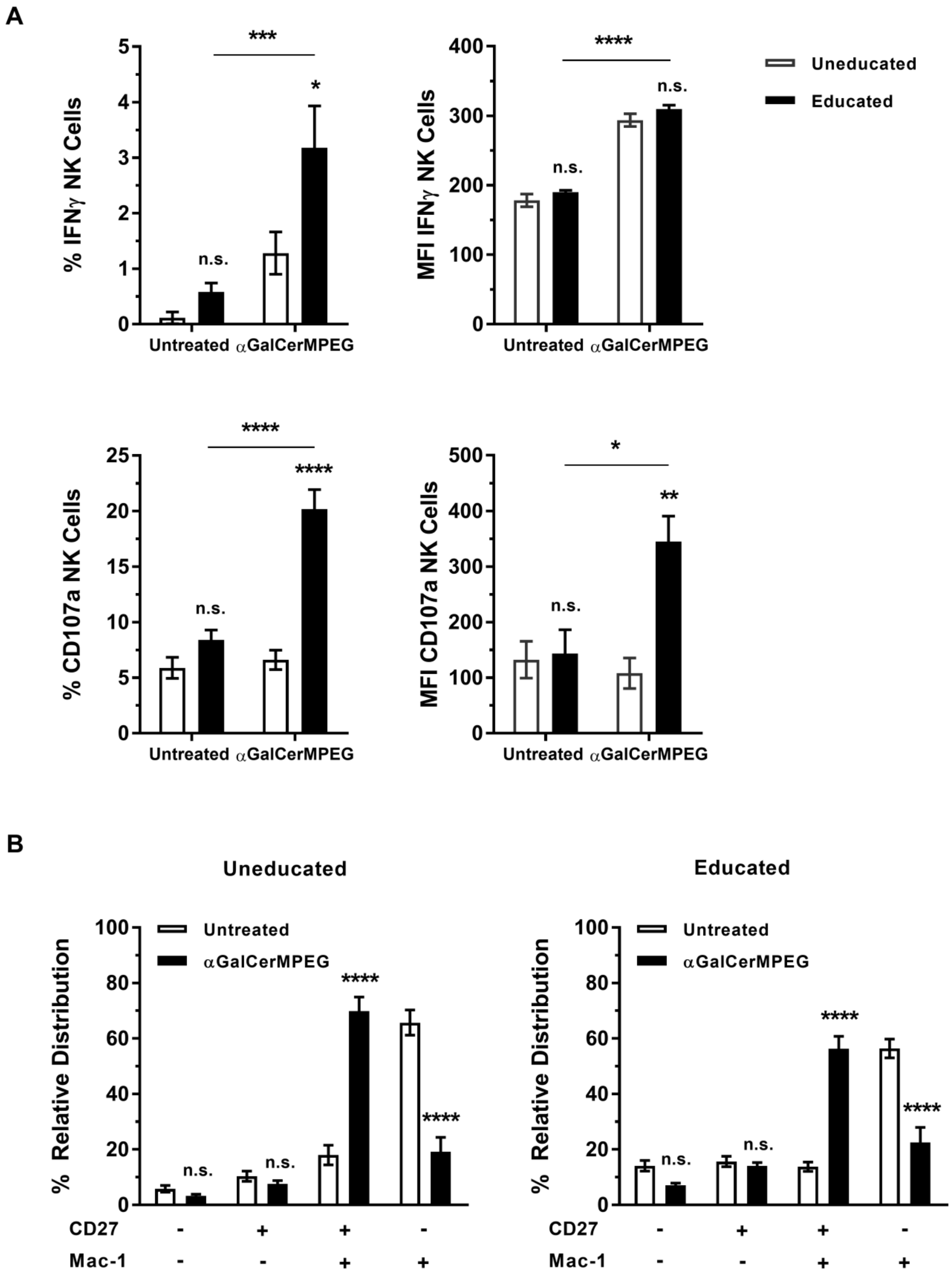

Figure S6. The impact of αGalCerMPEG administration on uneducated and educated NK cells in the liver. Wild type mice were injected by s.c. route with a single dose of αGalCerMPEG (10 µg) and hepatic lymphocytes were collected 72 h later. NK cells ($NKp46^{+}CD3^{-}$) were stained for CD27, Mac-1 uneducated ($Ly49C/I^{-}$ and $NKG2A^{-}$) and educated ($Ly49C/I^{+}$ or $NKG2A^{+}$) subsets and the expression of IFNγ and CD107a after 6 h co-incubation with YAC-1 target cells. (A) Frequency and MFI of IFNγ- and CD107a-expressing NK cells (MFI: n=4 mice, shown is one out of three independent experiments; Frequencies: n=11 mice). (B) Relative distribution of CD27 and Mac-1 expression within

the subset of uneducated and educated NK cells (n=12 mice, columns represent the mean ± SEM of data pooled from three independent experiments). Asterisks denote significant values as calculated by Two-way ANOVA as compared to untreated controls. **** $p \leq 0.0001$; *** $p \leq 0.001$; ** $p \leq 0.01$; * $p \leq 0.05$; n.s. = not significant.

Figure S7

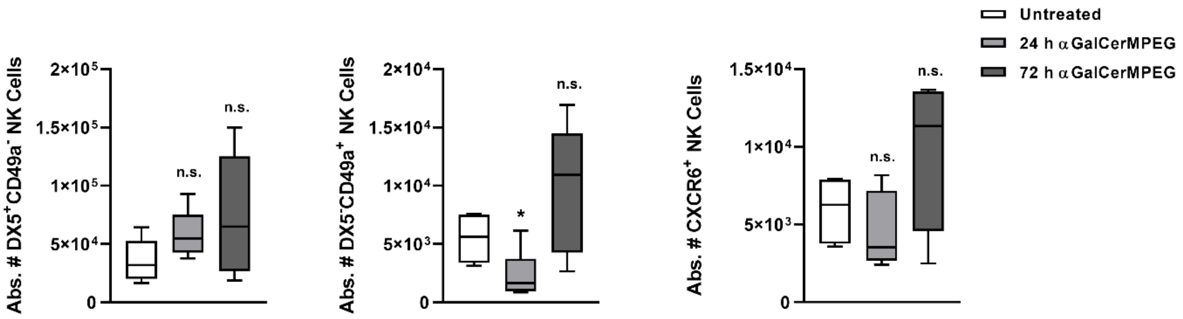

Figure S7. Absolute numbers of hepatic NK cell populations. Hepatic lymphocytes isolated from wt mice 24 and 72 h after administration of a single dose of αGalCerMPEG were assessed for the absolute numbers of hepatic NK cell populations (out of $1x10^6$ total cells) based on the surface expression of DX5, CD49a and CXCR6. Boxes represent the interquartile range, horizontal lines show the mean value and whiskers display the overall range of the data (n=6 mice, the shown data are derived from one experiment). Asterisks denote significant values as calculated by unpaired, two-tailed Student's t-test. * $p \leq 0.05$; n.s. = not significant.

Figure S8

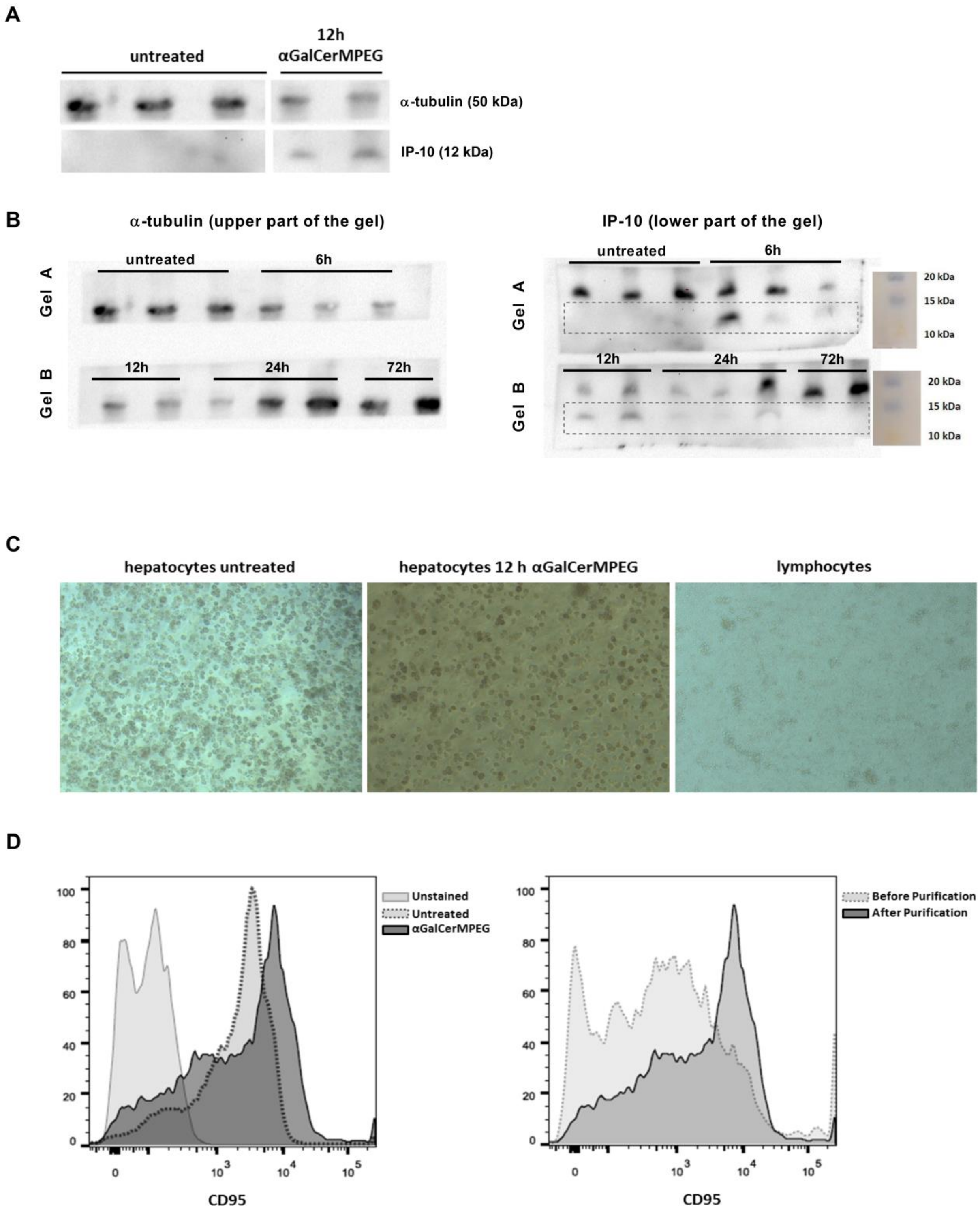

Figure S8. Assessment of IP-10 levels in hepatocytes.

Wild type mice were injected by s.c. route with a single dose of αGalCerMPEG (10 μg) and hepatocytes were isolated at the indicated time points for the assessment of IP-10 expression by western blot analysis. (A) Representative western blot membranes depict the bands detected for IP-10 and α-tubulin (loading control) in untreated and treated samples. (B) The shown membranes were cropped from different parts of two gels run simultaneously. The membranes were cut

for the incubation with the antibodies for α-tubulin (upper part of the gel) and IP-10 (lower part of the gel). (C) Representative light microscope pictures showing the isolated hepatocytes derived from untreated and treated samples and lymphocytes as reference (40x magnification). (D) Representative histograms for the flow cytometry analysis of CD95 expression on purified hepatocytes comparing untreated and treated samples (left) as well as before and after the purification steps (right).

Figure S9

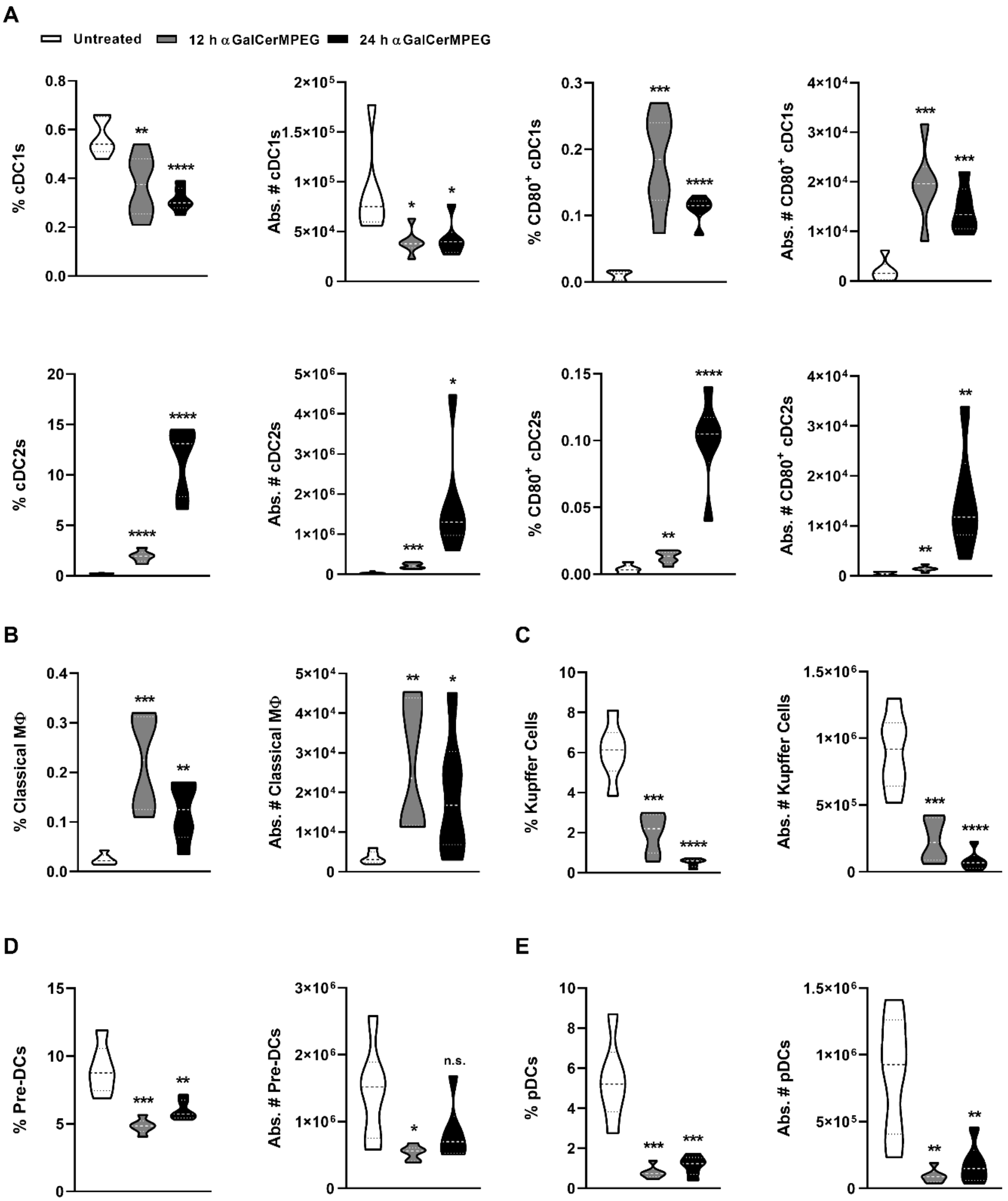

Figure S9. Impact of αGalCerMPEG administration on hepatic cell populations.

Wild type mice were injected by s.c. route with a single dose of αGalCerMPEG (10 µg) and hepatic cells were collected 12 h and 24 h later. The depicted hepatic cell populations were identified by flow cytometric analysis using the following cell surface markers: cDC1: CD45$^+$CD11c$^+$MHC cl. II$^+$PDCA1$^-$CD103$^+$CD11b$^-$CX3CR1$^-$F4/80$^-$, cDC2: CD45$^+$CD11c$^+$MHC cl. II$^+$PDCA1$^-$CD103$^-$CD11b$^+$CX3CR1$^+$Langerin$^-$, classical macrophages (MΦ): CD45$^+$F4/80$^+$CD11b^{high}Ly6C^{high}CX3CR1$^+$Ly6G$^+$, Kupffer cells: CD45$^+$CD11c$^-$F4/80+CD11b^{intermediate}, pDCs: CD45$^+$CD11c$^+$PDCA1$^+$, pre-DCs: CD45$^+$CD11c$^+$MHC cl. II$^-$. Violin plots represent the interquartile range, horizontal lines

show the mean value and the width displays the distribution of data points. n=6 mice, data derived from one experiment. Asterisks denote significant values as calculated by unpaired, two-tailed Student's t-test as compared to untreated controls. **** $p \leq 0.0001$;*** $p \leq 0.001$; ** $p \leq 0.01$; * $p \leq 0$; 05; n.s. = not significant.

Figure S10

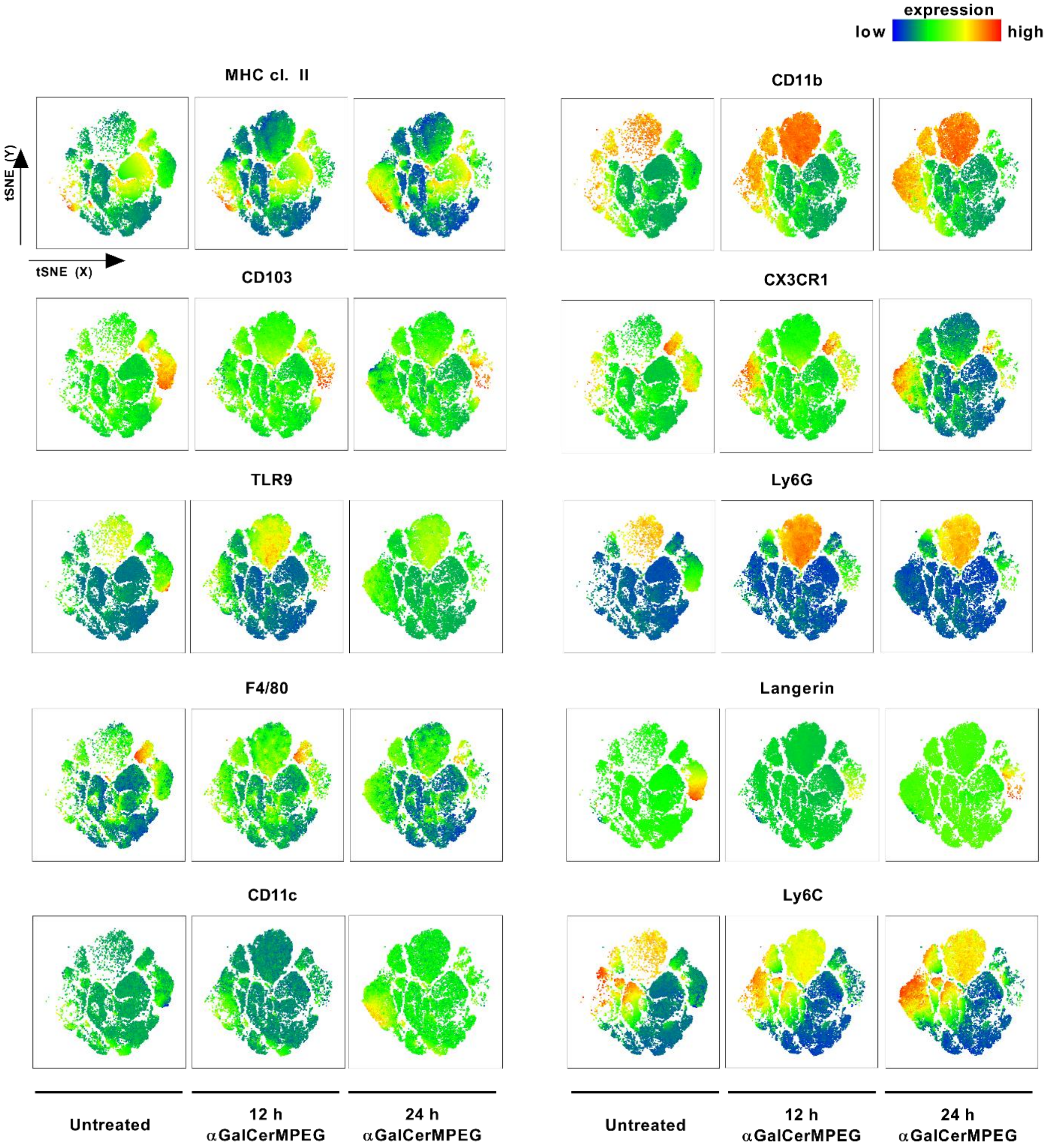

Figure S10. tSNE analysis of $CD45^+$ hepatic cell populations.
Wild type mice were injected by s.c. route with a single dose of αGalCerMPEG (10 µg) and hepatic cells were collected 12 and 24 h later. Cells were stained for CD45 and the indicated surface expression markers for subsequent analysis by flow cytometry. Shown is the tSNE analysis of $CD45^+$ cells for the indicated markers (blue=low expression, red=high expression).

Figure S11

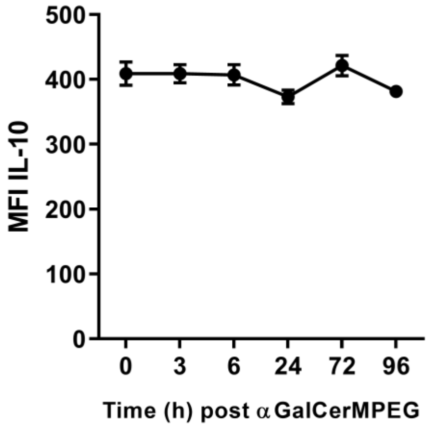

Figure S11. Serum IL-10 level upon αGalCerMPEG administration.
Serum samples were taken from wt mice at several time points after the administration of a single dose of αGalCerMPEG (10 µg) and IL-10 levels were assessed by cytometric bead array (MFI, n=5 mice).
